# (1*S*,3*R*)-*N*-{(3*S*,10*S*,12*S*,13*R*,17*R*)-12-Hy­droxy-17-[(*R*)-5-hy­droxy­pentan-2-yl]-10,13-di­methyl­hexa­deca­hydro-1*H*-cyclo­penta­[*a*]phenanthren-3-yl}adamantane-1-carboxamide 0.25-hydrate

**DOI:** 10.1107/S2414314622009476

**Published:** 2022-10-11

**Authors:** Cristian Campos Fernandez, R. Procupez-Schtirbu, V. H. Soto-Tellini, J. C. Salazar, Vojtech Jancik

**Affiliations:** a Escuela de Quimica, Universidad de Costa, Rica, San Jose, 2060, Costa Rica; b Escuela de Quimica, Laboratorio de Quimica Supramolecular, Universidad de Costa Rica, San Jose, 2060, Costa Rica; c Centro Conjunto de Investigacion en Quimica Sustentable UAE-UNAM, Carretera Toluca-Atlacomulco, km 14.5, Toluca, Mexico, 50200, Mexico; Goethe-Universität Frankfurt, Germany

**Keywords:** crystal structure, biological activity, bile salts

## Abstract

The title compound, C_35_H_57_NO_3_·0.25H_2_O, was synthesized from de­oxy­cholic acid followed by a protection, a Mitsonobu substitution, a Staudinger reduction, formation of an amide and final reduction in the lateral chain. The compound crystallizes in the *P*1 space group with four steroid mol­ecules and one water mol­ecule in the triclinic cell unit. The crystal structure features O—H⋯O hydrogen bonding. The crystal studied was refined as a non-merohedral twin.

## Abstract 2

The title compound was synthesized from de­oxy­cholic acid followed by a protection, a Mitsonobu substitution, a Staudinger reduction, formation of an amide and final reduction in the lateral chain.

## Graphical abstract

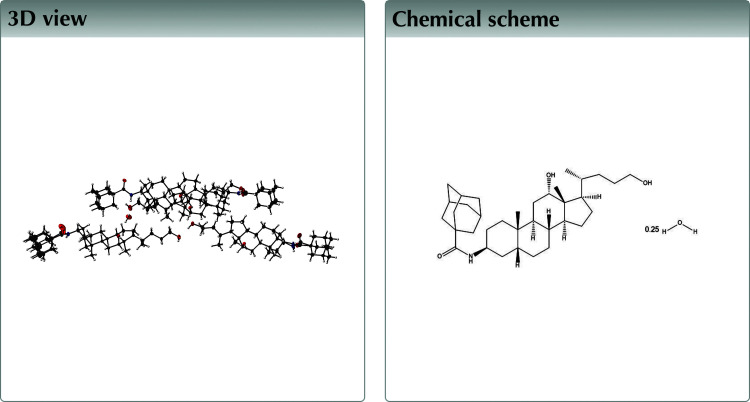

## Structure description

Bile salts are natural surfactants with a diverse biological activity, some of their deriv­atives show anti­proliferative, anti­microbial and anti­cancer activity (Huang *et al.*, 2009 ▸). Lately new compounds have been synthesized with a hydro­phobic expansion in the region 3β (Monte *et al.*, 2009 ▸); as a result, inter­esting supra­molecular properties arise and some of them show cytotoxic activity (Trillo *et al.*, 2014 ▸). Hydro­phobic derivatives of bile acids have been demonstrated to exhibit biological activity against certain cells; these derivatives cause apoptosis (programmed cell death), throughout a series of biochemical reactions inside the cellular body.

For inclusion crystals of 3α,7αα,24-tetra­hydroxy­cholane with aromatic compounds, see: Liu *et al.* (2013 ▸). For the inclusion abilities of cholic acid and its derivatives, see: Sada *et al.* (1994 ▸). For the lamellar structure formed by an adamantyl derivative of cholic acid, see: Soto *et al.* (2006 ▸).

**Figure 1:**
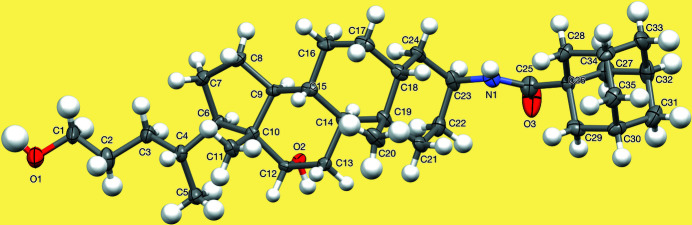
One of the four mol­ecules in the asymmetric unit of the title compound; disordered atoms (N1, O3, C25–C33) and the water mol­ecule are omitted for clarity.

**Figure 2:**
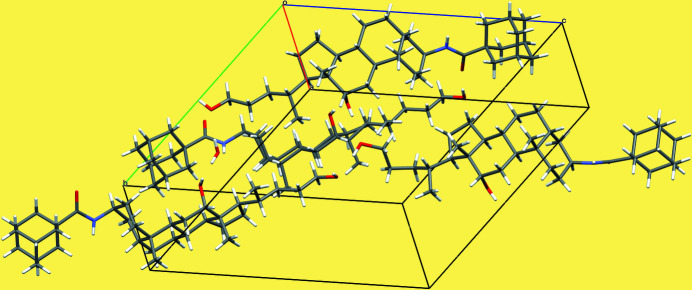
Packing diagram for the title compound.

There are four independent mol­ecules, which do not show any significant differences, and one water mol­ecule in the asymmetric unit of the title compound (Fig. 1 ▸), which crystallizes in space group *P*1. The crystal structure features extensive O—H⋯O hydrogen bonding (Table 1 ▸, Fig. 2 ▸), leading to the formation of a two-dimensional network parallel to (010).

## Synthesis and crystallization

The compound was initially obtained by the coupling of methyl 3β-amino­deoxy­cholate with adamantane-1-carbonyl chloride. Subsequently and after deprotecting the 24 position, the acid group was reduced with borane. The synthesis followed previously published methods (Trillo *et al.*, 2014 ▸).

## Refinement

Crystal data, data collection and structure refinement details are summarized in Table 2 ▸. The title compound crystallized as a non-merohedral twin (determined with *CELL_NOW*; Sheldrick, 2008*a* ▸) and an approximate ratio between the domains of 52:48% (determined with the refinement against hkl5 file). The data were integrated using the two orientation matrixes and *TWINABS* (Sheldrick, 2012 ▸) was used to generate the merged hkl4 file that contained only non-overlapped reflections belonging to the first domain and an hkl5 file containing single and overlapped reflections for both domains. As the refinement against the merged hkl4 file gave better results, it was selected over the hkl5 refinement. In case of H1*N*_1 and H1*N*′_1, FLAT 0.3 was used to keep the disordered protons close to the plane of the corresponding position of the disordered HN—C(O) peptide bond. The disordered groups (residue 1: HN—C(O), residue 4: adamantane carbonyl group) were refined using geometry (FLAT, SADI and SAME) and *U*_ij_ restraints (SIMU and RIGU) implemented in *SHELXL* (Sheldrick, 2015*b* ▸). Noteworthy, *Q*1 (0.32 e^−^ Å^−1^) corresponds to a second position of the OH group of residue 4 that does not modify appreciably the molecular conformation. However, as the occupancy is only about 8%, it was not refined.

## Supplementary Material

Crystal structure: contains datablock(s) I. DOI: 10.1107/S2414314622009476/bt4124sup1.cif

Structure factors: contains datablock(s) I. DOI: 10.1107/S2414314622009476/bt4124Isup7.hkl

Supporting information file. DOI: 10.1107/S2414314622009476/bt4124sup3.txt

Supporting information file. DOI: 10.1107/S2414314622009476/bt4124sup4.txt

Full stereochemistry diagram. DOI: 10.1107/S2414314622009476/bt4124sup5.pdf

CCDC reference: 1900390

Additional supporting information:  crystallographic information; 3D view; checkCIF report

## Figures and Tables

**Table 1:** Hydrogen-bond geometry (Å, °)

*D*—H⋯*A*	*D*—H	H⋯*A*	*D*⋯*A*	*D*—H⋯*A*
O1_1—H1_1⋯O1_4	0.88 (2)	1.85 (3)	2.717 (5)	172 (6)
O2_1—H2_1⋯O3_3^i^	0.87 (2)	2.02 (3)	2.882 (4)	170 (5)
O1_2—H1_2⋯O1_3^ii^	0.88 (2)	1.86 (4)	2.686 (5)	154 (7)
O2_2—H2_2⋯O1_1	0.87 (2)	1.93 (3)	2.795 (4)	171 (5)
C22_2—H22*A*_2⋯O3_2	0.99	2.64	3.159 (7)	113
O1_3—H1_3⋯O3_2	0.88 (2)	1.76 (3)	2.627 (5)	167 (6)
O2_3—H2_3⋯O2_2	0.87 (2)	2.05 (3)	2.913 (4)	173 (5)
O1_4—H1_4⋯O3_1^ii^	0.88 (2)	1.92 (3)	2.796 (6)	174 (6)
O1_4—H1_4⋯O3′_1^ii^	0.88 (2)	2.20 (5)	2.881 (14)	135 (5)
O2_4—H2_4⋯O2_1^ii^	0.74 (5)	2.16 (5)	2.869 (4)	163 (6)
C22_4—H22*A*_4⋯O10_4	0.99	2.58	3.152 (13)	116
O1*W*—H1*W*⋯O2_4	0.86 (2)	2.16 (2)	3.001 (5)	170 (7)
O1*W*—H2*W*⋯O1_2	0.86 (2)	1.93 (3)	2.762 (6)	165 (8)

Symmetry codes: (i) 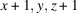 ; (ii) 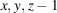 .

**Table 2:** Experimental details

Crystal data	
Chemical formula	C_35_H_57_NO_3_·0.25H_2_O
*M*_r_	544.32
Crystal system, space group	Triclinic, *P*1
Temperature (K)	100
*a*, *b*, *c* (Å)	11.8289 (6), 16.7287 (8), 17.7234 (9)
α, β, γ (°)	116.2071 (14), 91.0059 (15), 100.4537 (15)
*V* (Å^3^)	3075.4 (3)
*Z*	4
Radiation type	Mo *K*α
μ (mm^−1^)	0.07
Crystal size (mm)	0.50 × 0.30 × 0.20

Data collection	
Diffractometer	Bruker APEXII CCD
Absorption correction	Multi-scan (*TWINABS*; Sheldrick, 2012 ▸)
*T*_min_, *T*_max_	0.691, 0.745
No. of measured, independent and observed [*I* > 2σ(*I*)] reflections	22679, 22679, 18020
*R*_int_	0.049
(sin θ/λ)_max_ (Å^−1^)	0.606

Refinement	
*R*[*F* ^2^ > 2σ(*F* ^2^)], *wR*(*F* ^2^), *S*	0.062, 0.121, 1.07
No. of reflections	22679
No. of parameters	1612
No. of restraints	999
H-atom treatment	H atoms treated by a mixture of independent and constrained refinement
Δρ_max_, Δρ_min_ (e Å^−3^)	0.32, −0.25
Absolute structure	Flack *x* determined using 7166 quotients [(*I* ^+^)−(*I* ^−^)]/[(*I* ^+^)+(*I* ^−^)] (Parsons *et al.*, 2013 ▸)
Absolute structure parameter	0.2 (3)

Computer programs: *APEX2* and *SAINT* (Bruker, 2015 ▸), *SHELXT2014/5* (Sheldrick, 2015*a* ▸), *SHELXL2018/3* (Sheldrick, 2015*b* ▸), *SHELXTL* (Sheldrick, 2008*b* ▸).

## full crystallographic data

### Crystal data

**Table:**

C_35_H_57_NO_3_·0.25H_2_O	*Z* = 4
*M**_r_* = 544.32	*F*(000) = 1202
Triclinic, *P*1	*D*_x_ = 1.176 Mg m^−^^3^
*a* = 11.8289 (6) Å	Mo *K*α radiation, λ = 0.71073 Å
*b* = 16.7287 (8) Å	Cell parameters from 9865 reflections
*c* = 17.7234 (9) Å	θ = 2.6–25.4°
α = 116.2071 (14)°	µ = 0.07 mm^−^^1^
β = 91.0059 (15)°	*T* = 100 K
γ = 100.4537 (15)°	Block, pale yellow
*V* = 3075.4 (3) Å^3^	0.50 × 0.30 × 0.20 mm

### Data collection

**Table:**

Bruker APEXII CCD diffractometer	22679 independent reflections
Radiation source: sealed tube, Siemens KFFMO2K-90 with curved graphite	18020 reflections with *I* > 2σ(*I*)
Detector resolution: 10.4167 pixels mm^-1^	*R*_int_ = 0.049
φ and ω scans	θ_max_ = 25.5°, θ_min_ = 2.5°
Absorption correction: multi-scan (*TWINABS*; Sheldrick, 2012)	*h* = −14→14
*T*_min_ = 0.691, *T*_max_ = 0.745	*k* = −20→20
22679 measured reflections	*l* = −21→21

### Refinement

**Table:**

Refinement on *F*^2^	Secondary atom site location: difference Fourier map
Least-squares matrix: full	Hydrogen site location: mixed
*R*[*F*^2^ > 2σ(*F*^2^)] = 0.062	H atoms treated by a mixture of independent and constrained refinement
*wR*(*F*^2^) = 0.121	*w* = 1/[σ^2^(*F*_o_^2^) + (0.0371*P*)^2^ + 2.3114*P*] where *P* = (*F*_o_^2^ + 2*F*_c_^2^)/3
*S* = 1.07	(Δ/σ)_max_ < 0.001
22679 reflections	Δρ_max_ = 0.32 e Å^−^^3^
1612 parameters	Δρ_min_ = −0.25 e Å^−^^3^
999 restraints	Absolute structure: Flack *x* determined using 7166 quotients [(*I*^+^)-(*I*^-^)]/[(*I*^+^)+(*I*^-^)] (Parsons *et al.*, 2013)
Primary atom site location: structure-invariant direct methods	Absolute structure parameter: 0.2 (3)

### Special details

**Table:**

Geometry. All esds (except the esd in the dihedral angle between two l.s. planes) are estimated using the full covariance matrix. The cell esds are taken into account individually in the estimation of esds in distances, angles and torsion angles; correlations between esds in cell parameters are only used when they are defined by crystal symmetry. An approximate (isotropic) treatment of cell esds is used for estimating esds involving l.s. planes.
Refinement. Carbon-bound hydrogen atoms were placed in idealized positions and refined with a riding model. N-H hydrogen atoms were localized from the residual electron density map and refined with Uij tied to the parent atom with distance restraints.

### Fractional atomic coordinates and isotropic or equivalent isotropic displacement parameters (Å^2^)

**Table:**

	*x*	*y*	*z*	*U*_iso_*/*U*_eq_	Occ. (<1)
O1_1	0.5482 (3)	0.4623 (2)	0.4910 (2)	0.0333 (8)	
H1_1	0.599 (4)	0.503 (3)	0.484 (4)	0.050*	
O2_1	0.8067 (3)	0.54051 (19)	0.96446 (19)	0.0224 (7)	
H2_1	0.864 (3)	0.570 (3)	1.004 (2)	0.034*	
C23_1	0.7913 (5)	0.3837 (4)	1.1763 (3)	0.0395 (14)	
H23_1	0.754776	0.434464	1.214422	0.047*	
C25_1	0.8377 (7)	0.3765 (5)	1.3076 (4)	0.024 (2)	0.726 (8)
O3_1	0.8380 (4)	0.4588 (3)	1.3472 (3)	0.0347 (15)	0.726 (8)
N1_1	0.8200 (7)	0.3340 (5)	1.2232 (4)	0.024 (2)	0.726 (8)
H1N_1	0.816 (6)	0.278 (2)	1.197 (4)	0.029*	0.726 (8)
C26_1	0.8464 (4)	0.3129 (3)	1.3495 (3)	0.0230 (10)	
C25'_1	0.805 (2)	0.3598 (16)	1.3003 (11)	0.030 (4)	0.274 (8)
O3'_1	0.7242 (12)	0.3987 (10)	1.3260 (8)	0.047 (4)	0.274 (8)
N1'_1	0.8516 (19)	0.3530 (17)	1.2308 (11)	0.031 (5)	0.274 (8)
H1N'_1	0.905 (9)	0.326 (9)	1.213 (6)	0.037*	0.274 (8)
C1_1	0.5908 (4)	0.4629 (3)	0.5672 (3)	0.0239 (10)	
H1A_1	0.665090	0.442460	0.558841	0.029*	
H1B_1	0.535317	0.418589	0.578431	0.029*	
C2_1	0.6094 (4)	0.5549 (3)	0.6434 (3)	0.0258 (11)	
H2A_1	0.550257	0.587711	0.638148	0.031*	
H2B_1	0.686332	0.591176	0.645094	0.031*	
C3_1	0.6020 (4)	0.5472 (3)	0.7259 (3)	0.0250 (10)	
H3A_1	0.524955	0.510673	0.723497	0.030*	
H3B_1	0.608172	0.608969	0.773085	0.030*	
C4_1	0.6953 (4)	0.5035 (3)	0.7456 (3)	0.0210 (10)	
H4_1	0.706725	0.452365	0.691084	0.025*	
C5_1	0.8099 (4)	0.5730 (3)	0.7791 (3)	0.0284 (11)	
H5A_1	0.872834	0.541437	0.776992	0.043*	
H5B_1	0.806602	0.617124	0.837708	0.043*	
H5C_1	0.823728	0.604877	0.744119	0.043*	
C6_1	0.6547 (4)	0.4631 (3)	0.8049 (3)	0.0219 (10)	
H6_1	0.641545	0.514212	0.858800	0.026*	
C7_1	0.5379 (4)	0.3921 (3)	0.7687 (3)	0.0326 (12)	
H7A_1	0.472285	0.422298	0.789047	0.039*	
H7B_1	0.529312	0.363523	0.706119	0.039*	
C8_1	0.5392 (4)	0.3191 (3)	0.7995 (3)	0.0357 (13)	
H8A_1	0.468468	0.309758	0.826160	0.043*	
H8B_1	0.545736	0.260187	0.752137	0.043*	
C9_1	0.6466 (4)	0.3594 (3)	0.8641 (3)	0.0257 (11)	
H9_1	0.624720	0.407045	0.916736	0.031*	
C10_1	0.7331 (4)	0.4104 (3)	0.8285 (3)	0.0194 (10)	
C11_1	0.7771 (4)	0.3452 (3)	0.7475 (3)	0.0297 (11)	
H11A_1	0.712228	0.311280	0.702896	0.045*	
H11B_1	0.812053	0.302439	0.759109	0.045*	
H11C_1	0.835136	0.380488	0.728852	0.045*	
C12_1	0.8385 (4)	0.4657 (3)	0.8951 (3)	0.0176 (9)	
H12_1	0.899897	0.490533	0.868404	0.021*	
C13_1	0.8879 (4)	0.4060 (3)	0.9270 (3)	0.0183 (9)	
H13A_1	0.950891	0.445250	0.973086	0.022*	
H13B_1	0.922175	0.361560	0.880298	0.022*	
C14_1	0.7982 (4)	0.3538 (3)	0.9599 (3)	0.0186 (9)	
H14_1	0.766632	0.400676	1.007533	0.022*	
C15_1	0.6959 (4)	0.2962 (3)	0.8904 (3)	0.0267 (11)	
H15_1	0.726263	0.251550	0.840220	0.032*	
C16_1	0.6027 (4)	0.2434 (4)	0.9183 (4)	0.0412 (14)	
H16A_1	0.545693	0.200976	0.868951	0.049*	
H16B_1	0.561643	0.286458	0.960640	0.049*	
C17_1	0.6512 (4)	0.1888 (4)	0.9569 (4)	0.0410 (14)	
H17A_1	0.588391	0.160837	0.979436	0.049*	
H17B_1	0.681356	0.139049	0.912274	0.049*	
C18_1	0.7484 (4)	0.2502 (3)	1.0281 (3)	0.0274 (11)	
H18_1	0.780874	0.210169	1.047305	0.033*	
C19_1	0.8478 (4)	0.2969 (3)	0.9966 (3)	0.0203 (10)	
C20_1	0.9054 (4)	0.2249 (3)	0.9296 (3)	0.0323 (12)	
H20A_1	0.850147	0.187248	0.878925	0.048*	
H20B_1	0.928962	0.186169	0.952651	0.048*	
H20C_1	0.973496	0.255644	0.914709	0.048*	
C21_1	0.9412 (4)	0.3551 (3)	1.0719 (3)	0.0229 (10)	
H21A_1	0.972690	0.314447	1.089749	0.027*	
H21B_1	1.005277	0.386728	1.053327	0.027*	
C22_1	0.8969 (5)	0.4259 (3)	1.1477 (3)	0.0326 (12)	
H22A_1	0.876469	0.472297	1.132676	0.039*	
H22B_1	0.959475	0.457158	1.195320	0.039*	
C24_1	0.7015 (4)	0.3182 (4)	1.1034 (3)	0.0371 (13)	
H24A_1	0.660858	0.353826	1.084189	0.044*	
H24B_1	0.643645	0.284185	1.124206	0.044*	
C27_1	0.8646 (4)	0.3732 (3)	1.4442 (3)	0.0252 (10)	
H27A_1	0.797769	0.402533	1.462266	0.030*	
H27B_1	0.934853	0.421751	1.458670	0.030*	
C28_1	0.7377 (4)	0.2382 (3)	1.3280 (3)	0.0315 (12)	
H28A_1	0.724937	0.198487	1.266146	0.038*	
H28B_1	0.669566	0.266152	1.345149	0.038*	
C29_1	0.9514 (4)	0.2685 (3)	1.3226 (3)	0.0293 (11)	
H29A_1	0.941138	0.229597	1.260638	0.035*	
H29B_1	1.022332	0.316414	1.336837	0.035*	
C30_1	0.9640 (4)	0.2107 (3)	1.3681 (3)	0.0249 (11)	
H30_1	1.032237	0.181847	1.350082	0.030*	
C31_1	0.9827 (4)	0.2723 (3)	1.4634 (3)	0.0333 (12)	
H31A_1	1.053679	0.320206	1.477696	0.040*	
H31B_1	0.992764	0.236008	1.493626	0.040*	
C32_1	0.8781 (4)	0.3165 (3)	1.4915 (3)	0.0313 (12)	
H32_1	0.889730	0.356739	1.553915	0.038*	
C33_1	0.7690 (4)	0.2410 (4)	1.4686 (3)	0.0367 (13)	
H33A_1	0.777395	0.203955	1.498315	0.044*	
H33B_1	0.700801	0.268557	1.486832	0.044*	
C34_1	0.7516 (4)	0.1812 (3)	1.3744 (3)	0.0336 (12)	
H34_1	0.680294	0.132459	1.359800	0.040*	
C35_1	0.8556 (4)	0.1369 (3)	1.3465 (3)	0.0297 (11)	
H35A_1	0.864380	0.099400	1.375731	0.036*	
H35B_1	0.843580	0.096557	1.284799	0.036*	
O1_2	0.3373 (4)	0.4266 (3)	−0.0444 (2)	0.0534 (11)	
H1_2	0.292 (5)	0.405 (5)	−0.092 (3)	0.080*	
O2_2	0.3536 (2)	0.33539 (19)	0.38745 (18)	0.0201 (7)	
H2_2	0.409 (3)	0.377 (3)	0.424 (3)	0.030*	
O3_2	0.1866 (5)	0.1980 (3)	0.7377 (3)	0.0728 (15)	
N1_2	0.1928 (3)	0.0826 (3)	0.6147 (3)	0.0287 (9)	
H1N_2	0.196 (4)	0.028 (2)	0.588 (3)	0.034*	
C1_2	0.2741 (5)	0.3941 (4)	0.0063 (3)	0.0483 (16)	
H1A_2	0.219849	0.335840	−0.030024	0.058*	
H1B_2	0.228424	0.438611	0.041086	0.058*	
C2_2	0.3568 (5)	0.3794 (4)	0.0637 (3)	0.0384 (13)	
H2A_2	0.404858	0.337052	0.028693	0.046*	
H2B_2	0.409067	0.438245	0.101274	0.046*	
C3_2	0.2913 (4)	0.3407 (3)	0.1168 (3)	0.0294 (11)	
H3A_2	0.229172	0.288476	0.079391	0.035*	
H3B_2	0.253874	0.387754	0.158055	0.035*	
C4_2	0.3654 (4)	0.3087 (3)	0.1650 (3)	0.0238 (10)	
H4_2	0.408820	0.265830	0.123518	0.029*	
C5_2	0.4537 (4)	0.3898 (3)	0.2311 (3)	0.0314 (12)	
H5A_2	0.496062	0.424326	0.204077	0.047*	
H5B_2	0.508198	0.367594	0.255507	0.047*	
H5C_2	0.413453	0.429421	0.275910	0.047*	
C6_2	0.2886 (4)	0.2566 (3)	0.2047 (3)	0.0197 (10)	
H6_2	0.249190	0.301018	0.248537	0.024*	
C7_2	0.1921 (4)	0.1779 (3)	0.1393 (3)	0.0262 (11)	
H7A_2	0.121317	0.200893	0.136741	0.031*	
H7B_2	0.218559	0.153361	0.082212	0.031*	
C8_2	0.1665 (4)	0.1031 (3)	0.1682 (3)	0.0285 (11)	
H8A_2	0.083022	0.087471	0.172998	0.034*	
H8B_2	0.191397	0.047365	0.128359	0.034*	
C9_2	0.2374 (4)	0.1457 (3)	0.2548 (3)	0.0198 (10)	
H9_2	0.192101	0.187445	0.295503	0.024*	
C10_2	0.3455 (4)	0.2076 (3)	0.2474 (3)	0.0179 (9)	
C11_2	0.4273 (4)	0.1525 (3)	0.1884 (3)	0.0251 (10)	
H11A_2	0.388234	0.118157	0.130571	0.038*	
H11B_2	0.447877	0.110029	0.207920	0.038*	
H11C_2	0.497589	0.194279	0.189209	0.038*	
C12_2	0.4126 (3)	0.2661 (3)	0.3357 (3)	0.0181 (9)	
H12_2	0.490180	0.296399	0.329208	0.022*	
C13_2	0.4307 (4)	0.2072 (3)	0.3796 (3)	0.0201 (10)	
H13A_2	0.486414	0.169397	0.349997	0.024*	
H13B_2	0.466290	0.248168	0.438336	0.024*	
C14_2	0.3207 (4)	0.1441 (3)	0.3825 (3)	0.0196 (10)	
H14_2	0.266709	0.183954	0.413708	0.024*	
C15_2	0.2620 (4)	0.0833 (3)	0.2913 (3)	0.0205 (10)	
H15_2	0.317985	0.047449	0.257314	0.025*	
C16_2	0.1520 (4)	0.0159 (3)	0.2873 (3)	0.0263 (11)	
H16A_2	0.091550	0.050002	0.313718	0.032*	
H16B_2	0.122722	−0.025906	0.227364	0.032*	
C17_2	0.1746 (4)	−0.0396 (3)	0.3324 (3)	0.0302 (11)	
H17A_2	0.101035	−0.079416	0.331465	0.036*	
H17B_2	0.228292	−0.079112	0.302046	0.036*	
C18_2	0.2264 (4)	0.0215 (3)	0.4236 (3)	0.0253 (11)	
H18_2	0.245682	−0.018956	0.447692	0.030*	
C19_2	0.3411 (4)	0.0887 (3)	0.4306 (3)	0.0236 (10)	
C20_2	0.4347 (4)	0.0336 (3)	0.3936 (4)	0.0352 (13)	
H20A_2	0.417655	−0.000073	0.331868	0.053*	
H20B_2	0.435689	−0.009499	0.417056	0.053*	
H20C_2	0.510455	0.075177	0.408162	0.053*	
C21_2	0.3817 (4)	0.1503 (3)	0.5245 (3)	0.0288 (11)	
H21A_2	0.405048	0.112775	0.549874	0.035*	
H21B_2	0.451185	0.196116	0.529428	0.035*	
C22_2	0.2921 (4)	0.2004 (3)	0.5761 (3)	0.0320 (12)	
H22A_2	0.324027	0.235033	0.636387	0.038*	
H22B_2	0.276055	0.244575	0.556366	0.038*	
C23_2	0.1799 (4)	0.1351 (3)	0.5680 (3)	0.0286 (11)	
H23_2	0.120453	0.172036	0.593135	0.034*	
C24_2	0.1369 (4)	0.0716 (3)	0.4759 (3)	0.0269 (11)	
H24A_2	0.110823	0.107449	0.449572	0.032*	
H24B_2	0.068861	0.025829	0.473164	0.032*	
C25_2	0.1938 (5)	0.1187 (3)	0.6983 (3)	0.0333 (12)	
C26_2	0.2088 (4)	0.0612 (3)	0.7429 (3)	0.0244 (10)	
C27_2	0.1450 (5)	0.0927 (4)	0.8218 (3)	0.0352 (12)	
H27A_2	0.060870	0.079675	0.804918	0.042*	
H27B_2	0.171277	0.159356	0.856689	0.042*	
C28_2	0.1693 (4)	−0.0417 (3)	0.6897 (3)	0.0298 (11)	
H28A_2	0.212025	−0.062429	0.639312	0.036*	
H28B_2	0.085748	−0.056881	0.670082	0.036*	
C29_2	0.3398 (4)	0.0824 (3)	0.7717 (3)	0.0324 (12)	
H29A_2	0.383025	0.062845	0.721457	0.039*	
H29B_2	0.367376	0.148907	0.806562	0.039*	
C30_2	0.3623 (4)	0.0324 (3)	0.8233 (3)	0.0327 (12)	
H30_2	0.447117	0.046199	0.841110	0.039*	
C31_2	0.2980 (5)	0.0652 (4)	0.9010 (3)	0.0431 (14)	
H31A_2	0.325011	0.131788	0.935445	0.052*	
H31B_2	0.313950	0.035038	0.935912	0.052*	
C32_2	0.1683 (5)	0.0430 (4)	0.8743 (3)	0.0413 (14)	
H32_2	0.125818	0.063618	0.925643	0.050*	
C33_2	0.1265 (5)	−0.0593 (4)	0.8193 (4)	0.0429 (14)	
H33A_2	0.139505	−0.092244	0.852280	0.052*	
H33B_2	0.042560	−0.073205	0.801614	0.052*	
C34_2	0.1912 (4)	−0.0905 (3)	0.7423 (3)	0.0324 (12)	
H34_2	0.163807	−0.157742	0.707061	0.039*	
C35_2	0.3202 (4)	−0.0696 (3)	0.7698 (3)	0.0347 (12)	
H35A_2	0.334867	−0.101936	0.802965	0.042*	
H35B_2	0.362745	−0.091074	0.719308	0.042*	
O1_3	0.1496 (3)	0.3589 (2)	0.8414 (2)	0.0377 (9)	
H1_3	0.171 (5)	0.310 (3)	0.805 (3)	0.057*	
O2_3	0.2094 (3)	0.4679 (2)	0.41643 (19)	0.0230 (7)	
H2_3	0.252 (4)	0.428 (3)	0.403 (3)	0.034*	
O3_3	0.0159 (3)	0.6303 (2)	0.0795 (2)	0.0304 (8)	
N1_3	0.1909 (3)	0.6626 (3)	0.1498 (3)	0.0285 (9)	
H1N_3	0.262 (3)	0.676 (3)	0.151 (3)	0.034*	
C1_3	0.1007 (4)	0.3840 (3)	0.7836 (3)	0.0278 (11)	
H1A_3	0.055968	0.431089	0.814447	0.033*	
H1B_3	0.045923	0.330094	0.740359	0.033*	
C2_3	0.1883 (4)	0.4201 (3)	0.7398 (3)	0.0225 (10)	
H2A_3	0.231824	0.372746	0.707226	0.027*	
H2B_3	0.244026	0.473673	0.782544	0.027*	
C3_3	0.1296 (4)	0.4475 (3)	0.6801 (3)	0.0221 (10)	
H3A_3	0.085896	0.391499	0.631641	0.027*	
H3B_3	0.072875	0.483873	0.710397	0.027*	
C4_3	0.2104 (4)	0.5021 (3)	0.6458 (3)	0.0197 (10)	
H4_3	0.253506	0.558988	0.694880	0.024*	
C5_3	0.2988 (4)	0.4467 (3)	0.5977 (3)	0.0276 (11)	
H5A_3	0.259202	0.393730	0.546065	0.041*	
H5B_3	0.336084	0.426157	0.633703	0.041*	
H5C_3	0.357507	0.485067	0.582610	0.041*	
C6_3	0.1429 (3)	0.5299 (3)	0.5896 (3)	0.0182 (9)	
H6_3	0.104706	0.472364	0.539012	0.022*	
C7_3	0.0453 (4)	0.5790 (3)	0.6330 (3)	0.0213 (10)	
H7A_3	−0.027102	0.533964	0.623812	0.026*	
H7B_3	0.067925	0.615679	0.694650	0.026*	
C8_3	0.0276 (4)	0.6412 (3)	0.5924 (3)	0.0223 (10)	
H8A_3	−0.053916	0.627087	0.568483	0.027*	
H8B_3	0.048531	0.706143	0.634718	0.027*	
C9_3	0.1078 (3)	0.6208 (3)	0.5227 (3)	0.0190 (9)	
H9_3	0.065750	0.564043	0.472914	0.023*	
C10_3	0.2099 (4)	0.5943 (3)	0.5560 (3)	0.0176 (9)	
C11_3	0.2896 (4)	0.6752 (3)	0.6284 (3)	0.0218 (10)	
H11A_3	0.356310	0.655394	0.642830	0.033*	
H11B_3	0.247307	0.699474	0.677908	0.033*	
H11C_3	0.316510	0.722806	0.610887	0.033*	
C12_3	0.2792 (4)	0.5509 (3)	0.4811 (3)	0.0195 (10)	
H12_3	0.348704	0.536662	0.501448	0.023*	
C13_3	0.3190 (4)	0.6165 (3)	0.4433 (3)	0.0219 (10)	
H13A_3	0.358297	0.585202	0.392776	0.026*	
H13B_3	0.376241	0.669502	0.485170	0.026*	
C14_3	0.2203 (4)	0.6510 (3)	0.4176 (3)	0.0190 (9)	
H14_3	0.168447	0.596058	0.371806	0.023*	
C15_3	0.1456 (4)	0.6910 (3)	0.4902 (3)	0.0199 (10)	
H15_3	0.193561	0.747382	0.537162	0.024*	
C16_3	0.0416 (4)	0.7157 (3)	0.4591 (3)	0.0227 (10)	
H16A_3	−0.010909	0.659097	0.417531	0.027*	
H16B_3	−0.001488	0.746123	0.507565	0.027*	
C17_3	0.0776 (4)	0.7786 (3)	0.4181 (3)	0.0246 (10)	
H17A_3	0.119619	0.838783	0.462010	0.030*	
H17B_3	0.007357	0.787563	0.394321	0.030*	
C18_3	0.1549 (4)	0.7406 (3)	0.3475 (3)	0.0227 (10)	
H18_3	0.184545	0.789842	0.330944	0.027*	
C19_3	0.2617 (4)	0.7165 (3)	0.3784 (3)	0.0213 (10)	
C20_3	0.3431 (4)	0.8042 (3)	0.4429 (3)	0.0344 (12)	
H20A_3	0.413697	0.789437	0.458301	0.052*	
H20B_3	0.304487	0.833131	0.493549	0.052*	
H20C_3	0.363125	0.846155	0.417918	0.052*	
C21_3	0.3280 (4)	0.6724 (3)	0.3019 (3)	0.0308 (12)	
H21A_3	0.357861	0.718138	0.281933	0.037*	
H21B_3	0.395489	0.655603	0.320570	0.037*	
C22_3	0.2577 (4)	0.5892 (3)	0.2289 (3)	0.0322 (12)	
H22A_3	0.232092	0.541300	0.247137	0.039*	
H22B_3	0.306477	0.565392	0.182473	0.039*	
C23_3	0.1518 (4)	0.6102 (3)	0.1964 (3)	0.0277 (11)	
H23_3	0.100374	0.551156	0.156025	0.033*	
C24_3	0.0836 (4)	0.6602 (3)	0.2695 (3)	0.0251 (10)	
H24A_3	0.024911	0.682618	0.247956	0.030*	
H24B_3	0.041615	0.615590	0.287250	0.030*	
C25_3	0.1205 (4)	0.6638 (3)	0.0908 (3)	0.0222 (10)	
C26_3	0.1742 (4)	0.7068 (3)	0.0363 (3)	0.0205 (10)	
C27_3	0.1091 (4)	0.7802 (3)	0.0407 (3)	0.0333 (12)	
H27A_3	0.025362	0.753202	0.024799	0.040*	
H27B_3	0.119903	0.829778	0.099322	0.040*	
C28_3	0.3039 (4)	0.7502 (4)	0.0600 (3)	0.0333 (12)	
H28A_3	0.317185	0.799584	0.118832	0.040*	
H28B_3	0.347456	0.703737	0.056911	0.040*	
C29_3	0.1569 (5)	0.6317 (3)	−0.0551 (3)	0.0327 (12)	
H29A_3	0.198928	0.584087	−0.059393	0.039*	
H29B_3	0.073614	0.603062	−0.071894	0.039*	
C30_3	0.2011 (5)	0.6708 (3)	−0.1151 (3)	0.0367 (13)	
H30_3	0.189233	0.620939	−0.174467	0.044*	
C31_3	0.1360 (5)	0.7429 (4)	−0.1093 (3)	0.0413 (14)	
H31A_3	0.052512	0.715405	−0.126078	0.050*	
H31B_3	0.163943	0.767832	−0.148523	0.050*	
C32_3	0.1542 (4)	0.8191 (4)	−0.0194 (4)	0.0378 (13)	
H32_3	0.111191	0.866613	−0.015986	0.045*	
C33_3	0.2826 (5)	0.8617 (3)	0.0062 (3)	0.0389 (13)	
H33A_3	0.312143	0.888237	−0.031591	0.047*	
H33B_3	0.294947	0.911160	0.064930	0.047*	
C34_3	0.3470 (4)	0.7887 (4)	−0.0004 (3)	0.0351 (13)	
H34_3	0.431394	0.816588	0.015877	0.042*	
C35_3	0.3295 (5)	0.7135 (4)	−0.0895 (3)	0.0399 (13)	
H35A_3	0.372606	0.666614	−0.093227	0.048*	
H35B_3	0.359429	0.738274	−0.128384	0.048*	
O1_4	0.7055 (3)	0.5760 (3)	0.4538 (2)	0.0421 (9)	
H1_4	0.750 (5)	0.542 (4)	0.420 (3)	0.063*	
O2_4	0.6746 (3)	0.6801 (2)	0.0285 (2)	0.0244 (8)	
H2_4	0.696 (5)	0.638 (4)	0.010 (4)	0.037*	
C1_4	0.6439 (4)	0.6119 (4)	0.4086 (3)	0.0333 (12)	
H1A_4	0.607055	0.659849	0.449261	0.040*	0.99 (6)
H1B_4	0.581809	0.562467	0.367656	0.040*	0.99 (6)
C2_4	0.7221 (4)	0.6512 (3)	0.3621 (3)	0.0273 (11)	
H2A_4	0.764249	0.604815	0.325330	0.033*	
H2B_4	0.780002	0.704009	0.403627	0.033*	
C3_4	0.6567 (4)	0.6820 (3)	0.3075 (3)	0.0206 (10)	
H3A_4	0.619818	0.731924	0.344907	0.025*	
H3B_4	0.594498	0.630511	0.269284	0.025*	
C4_4	0.7349 (4)	0.7153 (3)	0.2540 (3)	0.0193 (10)	
H4_4	0.804748	0.759640	0.291836	0.023*	
C5_4	0.7748 (4)	0.6340 (3)	0.1858 (3)	0.0287 (11)	
H5A_4	0.708730	0.593003	0.144371	0.043*	
H5B_4	0.807257	0.601067	0.211919	0.043*	
H5C_4	0.834043	0.655657	0.157237	0.043*	
C6_4	0.6735 (3)	0.7639 (3)	0.2144 (3)	0.0182 (9)	
H6_4	0.607572	0.717376	0.173559	0.022*	
C7_4	0.6218 (4)	0.8417 (3)	0.2803 (3)	0.0223 (10)	
H7A_4	0.670085	0.871103	0.335554	0.027*	
H7B_4	0.542489	0.817514	0.288226	0.027*	
C8_4	0.6197 (4)	0.9115 (3)	0.2449 (3)	0.0210 (10)	
H8A_4	0.540558	0.921910	0.240691	0.025*	
H8B_4	0.672377	0.970482	0.281462	0.025*	
C9_4	0.6608 (4)	0.8670 (3)	0.1571 (3)	0.0182 (9)	
H9_4	0.591982	0.822342	0.118226	0.022*	
C10_4	0.7445 (3)	0.8101 (3)	0.1664 (3)	0.0161 (9)	
C11_4	0.8583 (4)	0.8703 (3)	0.2209 (3)	0.0211 (10)	
H11A_4	0.911342	0.831579	0.220828	0.032*	
H11B_4	0.842415	0.906637	0.278949	0.032*	
H11C_4	0.893558	0.910957	0.197518	0.032*	
C12_4	0.7735 (4)	0.7506 (3)	0.0776 (3)	0.0184 (9)	
H12_4	0.837163	0.720882	0.083299	0.022*	
C13_4	0.8147 (4)	0.8068 (3)	0.0310 (3)	0.0207 (10)	
H13A_4	0.824805	0.764479	−0.027613	0.025*	
H13B_4	0.891583	0.845745	0.058845	0.025*	
C14_4	0.7343 (4)	0.8680 (3)	0.0272 (3)	0.0180 (9)	
H14_4	0.659405	0.826670	−0.004885	0.022*	
C15_4	0.7082 (4)	0.9283 (3)	0.1170 (3)	0.0172 (9)	
H15_4	0.782578	0.968753	0.150881	0.021*	
C16_4	0.6258 (4)	0.9887 (3)	0.1163 (3)	0.0225 (10)	
H16A_4	0.548608	0.950049	0.089045	0.027*	
H16B_4	0.617473	1.031114	0.175273	0.027*	
C17_4	0.6692 (4)	1.0436 (3)	0.0689 (3)	0.0250 (10)	
H17A_4	0.740967	1.088464	0.100760	0.030*	
H17B_4	0.610481	1.077768	0.066047	0.030*	
C18_4	0.6935 (4)	0.9829 (3)	−0.0207 (3)	0.0203 (10)	
H18_4	0.731737	1.024503	−0.043795	0.024*	
C19_4	0.7797 (4)	0.9236 (3)	−0.0210 (3)	0.0212 (10)	
C20_4	0.8990 (4)	0.9863 (3)	0.0208 (3)	0.0299 (11)	
H20A_4	0.957032	0.949000	0.012724	0.045*	
H20B_4	0.895442	1.022509	0.081496	0.045*	
H20C_4	0.920226	1.027034	−0.005163	0.045*	
C21_4	0.7930 (4)	0.8617 (3)	−0.1132 (3)	0.0289 (11)	
H21A_4	0.831200	0.899899	−0.138942	0.035*	
H21B_4	0.844308	0.820584	−0.114429	0.035*	
C22_4	0.6790 (5)	0.8040 (3)	−0.1669 (3)	0.0357 (13)	
H22A_4	0.694269	0.766053	−0.225473	0.043*	
H22B_4	0.642427	0.762757	−0.143753	0.043*	
C23_4	0.5967 (4)	0.8636 (3)	−0.1680 (3)	0.0305 (12)	
H23_4	0.519976	0.823394	−0.197360	0.037*	
C24_4	0.5806 (4)	0.9279 (3)	−0.0781 (3)	0.0244 (10)	
H24A_4	0.535765	0.891844	−0.052704	0.029*	
H24B_4	0.534384	0.970838	−0.079990	0.029*	
N1_4	0.6394 (3)	0.9150 (3)	−0.2140 (2)	0.0265 (9)	
H1N_4	0.676 (4)	0.969 (2)	−0.190 (3)	0.032*	
C25_4	0.6397 (12)	0.8718 (7)	−0.3001 (5)	0.033 (3)	0.511 (5)
O10_4	0.5972 (14)	0.7907 (6)	−0.3431 (6)	0.071 (5)	0.511 (5)
C26_4	0.6906 (8)	0.9295 (6)	−0.3434 (5)	0.027 (2)	0.511 (5)
C27_4	0.6106 (6)	0.9076 (6)	−0.4215 (5)	0.0320 (19)	0.511 (5)
H27A_4	0.596546	0.841379	−0.459805	0.038*	0.511 (5)
H27B_4	0.535287	0.923205	−0.404080	0.038*	0.511 (5)
C28_4	0.7161 (8)	1.0325 (5)	−0.2844 (5)	0.042 (2)	0.511 (5)
H28A_4	0.769712	1.046528	−0.234567	0.051*	0.511 (5)
H28B_4	0.643416	1.051594	−0.264129	0.051*	0.511 (5)
C29_4	0.8054 (6)	0.9033 (6)	−0.3723 (5)	0.036 (2)	0.511 (5)
H29A_4	0.858859	0.917399	−0.322427	0.043*	0.511 (5)
H29B_4	0.791741	0.836868	−0.409714	0.043*	0.511 (5)
C30_4	0.8607 (8)	0.9547 (7)	−0.4198 (6)	0.040 (2)	0.511 (5)
H30_4	0.935086	0.936182	−0.438761	0.049*	0.511 (5)
C31_4	0.7771 (7)	0.9335 (6)	−0.4966 (5)	0.035 (2)	0.511 (5)
H31A_4	0.812072	0.967752	−0.526918	0.042*	0.511 (5)
H31B_4	0.763082	0.867661	−0.536109	0.042*	0.511 (5)
C32_4	0.6623 (8)	0.9601 (6)	−0.4684 (6)	0.034 (2)	0.511 (5)
H32_4	0.607817	0.945872	−0.518584	0.041*	0.511 (5)
C33_4	0.6859 (9)	1.0622 (6)	−0.4077 (6)	0.047 (2)	0.511 (5)
H33A_4	0.717970	1.097240	−0.438002	0.056*	0.511 (5)
H33B_4	0.612344	1.079939	−0.387914	0.056*	0.511 (5)
C34_4	0.7708 (9)	1.0853 (6)	−0.3320 (6)	0.051 (3)	0.511 (5)
H34_4	0.786643	1.152290	−0.293656	0.061*	0.511 (5)
C35_4	0.8826 (8)	1.0561 (6)	−0.3626 (6)	0.053 (3)	0.511 (5)
H35A_4	0.917055	1.089426	−0.393872	0.064*	0.511 (5)
H35B_4	0.938416	1.071921	−0.313377	0.064*	0.511 (5)
C25'_4	0.6223 (12)	0.8760 (7)	−0.3004 (5)	0.032 (3)	0.489 (5)
O10'_4	0.5539 (15)	0.8043 (8)	−0.3426 (7)	0.092 (7)	0.489 (5)
C26'_4	0.6839 (8)	0.9309 (6)	−0.3432 (5)	0.029 (2)	0.489 (5)
C27'_4	0.6175 (7)	1.0021 (6)	−0.3400 (6)	0.045 (2)	0.489 (5)
H27C_4	0.537282	0.971748	−0.366828	0.053*	0.489 (5)
H27D_4	0.613978	1.045188	−0.280280	0.053*	0.489 (5)
C28'_4	0.8097 (6)	0.9781 (6)	−0.3035 (5)	0.035 (2)	0.489 (5)
H28C_4	0.810278	1.021702	−0.243381	0.042*	0.489 (5)
H28D_4	0.853274	0.931893	−0.305881	0.042*	0.489 (5)
C29'_4	0.6883 (7)	0.8643 (5)	−0.4352 (4)	0.029 (2)	0.489 (5)
H29C_4	0.608814	0.831295	−0.462193	0.035*	0.489 (5)
H29D_4	0.733174	0.818887	−0.437444	0.035*	0.489 (5)
C30'_4	0.7443 (8)	0.9144 (6)	−0.4837 (5)	0.034 (2)	0.489 (5)
H30'_4	0.744903	0.869887	−0.544109	0.041*	0.489 (5)
C31'_4	0.6753 (9)	0.9851 (6)	−0.4791 (6)	0.040 (3)	0.489 (5)
H31C_4	0.594816	0.954360	−0.504961	0.048*	0.489 (5)
H31D_4	0.710365	1.017384	−0.510997	0.048*	0.489 (5)
C32'_4	0.6753 (9)	1.0544 (7)	−0.3856 (6)	0.050 (3)	0.489 (5)
H32'_4	0.631926	1.101782	−0.381977	0.060*	0.489 (5)
C33'_4	0.8005 (8)	1.0992 (6)	−0.3455 (7)	0.049 (3)	0.489 (5)
H33C_4	0.837917	1.133706	−0.374809	0.059*	0.489 (5)
H33D_4	0.800823	1.142701	−0.285361	0.059*	0.489 (5)
C34'_4	0.8684 (8)	1.0288 (6)	−0.3513 (6)	0.041 (2)	0.489 (5)
H34'_4	0.949860	1.059317	−0.325285	0.049*	0.489 (5)
C35'_4	0.8669 (8)	0.9627 (7)	−0.4439 (6)	0.038 (3)	0.489 (5)
H35C_4	0.902641	0.996235	−0.474509	0.046*	0.489 (5)
H35D_4	0.913035	0.917138	−0.448653	0.046*	0.489 (5)
O1W	0.4487 (4)	0.6012 (3)	0.0687 (3)	0.0596 (12)	
H1W	0.509 (4)	0.628 (5)	0.057 (5)	0.089*	
H2W	0.425 (6)	0.547 (2)	0.028 (3)	0.089*	

### Atomic displacement parameters (Å^2^)

**Table:**

	*U*^11^	*U*^22^	*U*^33^	*U*^12^	*U*^13^	*U*^23^
O1_1	0.041 (2)	0.033 (2)	0.0234 (18)	−0.0054 (16)	−0.0084 (15)	0.0159 (16)
O2_1	0.0273 (17)	0.0161 (16)	0.0180 (17)	0.0055 (14)	−0.0025 (13)	0.0025 (14)
C23_1	0.073 (4)	0.033 (3)	0.034 (3)	0.036 (3)	0.028 (3)	0.023 (3)
C25_1	0.025 (4)	0.024 (4)	0.022 (3)	0.008 (3)	−0.001 (3)	0.007 (3)
O3_1	0.049 (3)	0.025 (3)	0.030 (3)	0.011 (2)	0.003 (2)	0.011 (2)
N1_1	0.045 (5)	0.014 (4)	0.021 (3)	0.008 (4)	0.013 (3)	0.013 (3)
C26_1	0.026 (2)	0.026 (2)	0.019 (2)	0.015 (2)	0.0051 (18)	0.009 (2)
C25'_1	0.038 (7)	0.031 (6)	0.023 (6)	0.018 (6)	0.008 (5)	0.010 (5)
O3'_1	0.053 (8)	0.063 (9)	0.045 (8)	0.040 (7)	0.017 (6)	0.030 (7)
N1'_1	0.048 (8)	0.016 (7)	0.028 (6)	0.006 (7)	0.020 (6)	0.009 (5)
C1_1	0.028 (2)	0.026 (3)	0.023 (3)	0.004 (2)	0.002 (2)	0.016 (2)
C2_1	0.034 (3)	0.020 (2)	0.024 (3)	0.001 (2)	−0.004 (2)	0.012 (2)
C3_1	0.035 (3)	0.017 (2)	0.021 (2)	0.006 (2)	−0.001 (2)	0.006 (2)
C4_1	0.031 (2)	0.017 (2)	0.015 (2)	0.006 (2)	−0.0004 (19)	0.007 (2)
C5_1	0.033 (3)	0.030 (3)	0.029 (3)	0.005 (2)	0.003 (2)	0.020 (2)
C6_1	0.024 (2)	0.019 (2)	0.020 (2)	−0.0002 (19)	−0.0050 (19)	0.008 (2)
C7_1	0.031 (3)	0.029 (3)	0.042 (3)	−0.004 (2)	−0.011 (2)	0.023 (3)
C8_1	0.029 (3)	0.031 (3)	0.049 (3)	−0.006 (2)	−0.010 (2)	0.025 (3)
C9_1	0.026 (2)	0.018 (2)	0.031 (3)	−0.003 (2)	−0.007 (2)	0.013 (2)
C10_1	0.026 (2)	0.014 (2)	0.017 (2)	0.0018 (19)	−0.0012 (18)	0.0077 (19)
C11_1	0.039 (3)	0.021 (2)	0.026 (3)	0.007 (2)	−0.006 (2)	0.008 (2)
C12_1	0.022 (2)	0.016 (2)	0.017 (2)	0.0071 (18)	0.0044 (18)	0.0086 (19)
C13_1	0.020 (2)	0.017 (2)	0.018 (2)	0.0017 (18)	0.0000 (17)	0.0089 (19)
C14_1	0.020 (2)	0.014 (2)	0.021 (2)	0.0037 (18)	0.0015 (18)	0.0083 (19)
C15_1	0.027 (2)	0.019 (2)	0.036 (3)	−0.001 (2)	−0.007 (2)	0.016 (2)
C16_1	0.027 (3)	0.044 (3)	0.063 (4)	−0.011 (2)	−0.013 (3)	0.041 (3)
C17_1	0.031 (3)	0.043 (3)	0.061 (4)	−0.007 (2)	−0.006 (3)	0.040 (3)
C18_1	0.029 (3)	0.024 (3)	0.038 (3)	0.004 (2)	−0.001 (2)	0.023 (2)
C19_1	0.023 (2)	0.016 (2)	0.024 (2)	0.0038 (19)	0.0012 (19)	0.011 (2)
C20_1	0.048 (3)	0.025 (3)	0.026 (3)	0.016 (2)	0.002 (2)	0.011 (2)
C21_1	0.026 (2)	0.025 (2)	0.024 (3)	0.004 (2)	−0.0006 (19)	0.017 (2)
C22_1	0.056 (3)	0.021 (3)	0.020 (3)	0.008 (2)	−0.002 (2)	0.009 (2)
C24_1	0.037 (3)	0.047 (3)	0.051 (4)	0.020 (3)	0.020 (3)	0.038 (3)
C27_1	0.029 (3)	0.019 (2)	0.027 (3)	0.007 (2)	0.001 (2)	0.010 (2)
C28_1	0.024 (2)	0.029 (3)	0.030 (3)	0.005 (2)	−0.007 (2)	0.002 (2)
C29_1	0.030 (3)	0.032 (3)	0.031 (3)	0.012 (2)	0.013 (2)	0.016 (2)
C30_1	0.018 (2)	0.024 (2)	0.034 (3)	0.010 (2)	0.009 (2)	0.012 (2)
C31_1	0.032 (3)	0.024 (3)	0.040 (3)	0.000 (2)	−0.013 (2)	0.014 (2)
C32_1	0.053 (3)	0.024 (3)	0.016 (2)	0.020 (2)	−0.002 (2)	0.004 (2)
C33_1	0.041 (3)	0.042 (3)	0.046 (3)	0.019 (3)	0.021 (3)	0.032 (3)
C34_1	0.017 (2)	0.026 (3)	0.053 (4)	0.001 (2)	0.005 (2)	0.015 (3)
C35_1	0.032 (3)	0.017 (2)	0.032 (3)	0.006 (2)	0.004 (2)	0.004 (2)
O1_2	0.065 (3)	0.054 (3)	0.037 (2)	−0.012 (2)	−0.006 (2)	0.027 (2)
O2_2	0.0246 (17)	0.0159 (16)	0.0168 (17)	0.0080 (13)	0.0005 (13)	0.0033 (14)
O3_2	0.160 (5)	0.034 (2)	0.041 (3)	0.053 (3)	0.029 (3)	0.020 (2)
N1_2	0.033 (2)	0.025 (2)	0.034 (3)	0.0148 (19)	0.0111 (19)	0.015 (2)
C1_2	0.061 (4)	0.053 (4)	0.028 (3)	−0.016 (3)	−0.009 (3)	0.027 (3)
C2_2	0.053 (3)	0.038 (3)	0.037 (3)	0.019 (3)	0.011 (3)	0.024 (3)
C3_2	0.038 (3)	0.029 (3)	0.022 (3)	0.006 (2)	0.000 (2)	0.013 (2)
C4_2	0.031 (2)	0.024 (2)	0.022 (2)	0.009 (2)	0.0054 (19)	0.013 (2)
C5_2	0.036 (3)	0.031 (3)	0.031 (3)	0.002 (2)	0.001 (2)	0.019 (2)
C6_2	0.022 (2)	0.020 (2)	0.015 (2)	0.0089 (19)	0.0018 (18)	0.0046 (19)
C7_2	0.028 (3)	0.027 (3)	0.020 (2)	0.006 (2)	−0.0004 (19)	0.008 (2)
C8_2	0.029 (3)	0.025 (3)	0.024 (3)	0.001 (2)	−0.005 (2)	0.006 (2)
C9_2	0.016 (2)	0.015 (2)	0.021 (2)	0.0026 (18)	0.0012 (18)	0.0031 (19)
C10_2	0.019 (2)	0.016 (2)	0.021 (2)	0.0084 (19)	0.0045 (18)	0.009 (2)
C11_2	0.029 (2)	0.025 (3)	0.028 (3)	0.012 (2)	0.010 (2)	0.016 (2)
C12_2	0.014 (2)	0.017 (2)	0.026 (2)	0.0023 (18)	0.0026 (18)	0.012 (2)
C13_2	0.019 (2)	0.021 (2)	0.021 (2)	0.0063 (19)	0.0026 (18)	0.009 (2)
C14_2	0.020 (2)	0.015 (2)	0.026 (3)	0.0098 (19)	0.0071 (19)	0.010 (2)
C15_2	0.021 (2)	0.013 (2)	0.026 (2)	0.0063 (19)	0.0050 (19)	0.006 (2)
C16_2	0.024 (2)	0.016 (2)	0.034 (3)	0.000 (2)	−0.001 (2)	0.010 (2)
C17_2	0.031 (3)	0.017 (2)	0.043 (3)	0.006 (2)	0.008 (2)	0.014 (2)
C18_2	0.025 (2)	0.020 (2)	0.041 (3)	0.010 (2)	0.010 (2)	0.020 (2)
C19_2	0.024 (2)	0.024 (2)	0.033 (3)	0.011 (2)	0.009 (2)	0.019 (2)
C20_2	0.029 (3)	0.040 (3)	0.057 (4)	0.019 (2)	0.017 (2)	0.035 (3)
C21_2	0.026 (2)	0.032 (3)	0.039 (3)	0.004 (2)	0.001 (2)	0.026 (2)
C22_2	0.047 (3)	0.030 (3)	0.025 (3)	0.012 (2)	0.003 (2)	0.016 (2)
C23_2	0.034 (3)	0.032 (3)	0.036 (3)	0.021 (2)	0.015 (2)	0.024 (2)
C24_2	0.021 (2)	0.032 (3)	0.040 (3)	0.009 (2)	0.008 (2)	0.026 (2)
C25_2	0.045 (3)	0.025 (3)	0.031 (3)	0.014 (2)	0.007 (2)	0.011 (2)
C26_2	0.030 (3)	0.023 (2)	0.024 (3)	0.009 (2)	0.004 (2)	0.013 (2)
C27_2	0.043 (3)	0.033 (3)	0.033 (3)	0.016 (2)	0.010 (2)	0.016 (3)
C28_2	0.040 (3)	0.024 (3)	0.024 (3)	0.009 (2)	−0.004 (2)	0.010 (2)
C29_2	0.034 (3)	0.023 (3)	0.044 (3)	0.003 (2)	0.004 (2)	0.020 (2)
C30_2	0.031 (3)	0.028 (3)	0.038 (3)	0.004 (2)	−0.006 (2)	0.016 (2)
C31_2	0.061 (4)	0.035 (3)	0.030 (3)	0.009 (3)	−0.008 (3)	0.012 (3)
C32_2	0.053 (3)	0.051 (4)	0.029 (3)	0.024 (3)	0.017 (3)	0.021 (3)
C33_2	0.040 (3)	0.052 (4)	0.054 (4)	0.002 (3)	0.002 (3)	0.042 (3)
C34_2	0.039 (3)	0.017 (2)	0.037 (3)	−0.003 (2)	−0.008 (2)	0.012 (2)
C35_2	0.046 (3)	0.025 (3)	0.036 (3)	0.012 (2)	−0.003 (2)	0.015 (2)
O1_3	0.052 (2)	0.040 (2)	0.031 (2)	0.0183 (18)	0.0081 (17)	0.0210 (18)
O2_3	0.0303 (17)	0.0170 (16)	0.0206 (17)	0.0109 (14)	−0.0003 (13)	0.0055 (14)
O3_3	0.0237 (18)	0.039 (2)	0.0245 (18)	−0.0006 (16)	0.0034 (14)	0.0139 (16)
N1_3	0.027 (2)	0.031 (2)	0.033 (2)	0.0017 (19)	0.0015 (19)	0.022 (2)
C1_3	0.034 (3)	0.032 (3)	0.024 (3)	0.012 (2)	0.003 (2)	0.016 (2)
C2_3	0.025 (2)	0.022 (2)	0.021 (2)	0.008 (2)	0.0033 (19)	0.010 (2)
C3_3	0.023 (2)	0.023 (2)	0.018 (2)	0.008 (2)	0.0023 (18)	0.007 (2)
C4_3	0.024 (2)	0.018 (2)	0.017 (2)	0.0051 (19)	0.0001 (18)	0.007 (2)
C5_3	0.026 (2)	0.036 (3)	0.032 (3)	0.012 (2)	0.007 (2)	0.023 (2)
C6_3	0.019 (2)	0.017 (2)	0.015 (2)	0.0033 (19)	0.0000 (17)	0.0040 (19)
C7_3	0.019 (2)	0.023 (2)	0.019 (2)	0.0052 (19)	−0.0002 (18)	0.006 (2)
C8_3	0.020 (2)	0.019 (2)	0.028 (3)	0.0063 (19)	0.0016 (19)	0.009 (2)
C9_3	0.020 (2)	0.013 (2)	0.021 (2)	0.0050 (18)	−0.0002 (18)	0.0042 (19)
C10_3	0.019 (2)	0.016 (2)	0.018 (2)	0.0037 (18)	0.0006 (18)	0.0077 (19)
C11_3	0.024 (2)	0.017 (2)	0.023 (3)	0.0036 (19)	−0.0011 (19)	0.008 (2)
C12_3	0.020 (2)	0.020 (2)	0.021 (2)	0.0079 (19)	0.0023 (18)	0.010 (2)
C13_3	0.020 (2)	0.024 (2)	0.025 (3)	0.008 (2)	0.0055 (19)	0.013 (2)
C14_3	0.020 (2)	0.014 (2)	0.024 (2)	0.0047 (18)	−0.0007 (18)	0.009 (2)
C15_3	0.019 (2)	0.015 (2)	0.023 (2)	0.0020 (18)	−0.0014 (18)	0.008 (2)
C16_3	0.021 (2)	0.023 (2)	0.026 (3)	0.008 (2)	0.0037 (19)	0.011 (2)
C17_3	0.025 (2)	0.019 (2)	0.032 (3)	0.008 (2)	−0.001 (2)	0.013 (2)
C18_3	0.020 (2)	0.019 (2)	0.035 (3)	0.0029 (19)	−0.001 (2)	0.018 (2)
C19_3	0.019 (2)	0.020 (2)	0.029 (3)	0.0013 (19)	−0.0001 (19)	0.016 (2)
C20_3	0.028 (3)	0.032 (3)	0.052 (3)	−0.001 (2)	−0.004 (2)	0.030 (3)
C21_3	0.025 (2)	0.044 (3)	0.043 (3)	0.013 (2)	0.009 (2)	0.034 (3)
C22_3	0.046 (3)	0.031 (3)	0.037 (3)	0.019 (2)	0.021 (2)	0.025 (3)
C23_3	0.035 (3)	0.025 (3)	0.026 (3)	−0.001 (2)	0.001 (2)	0.018 (2)
C24_3	0.024 (2)	0.024 (2)	0.030 (3)	0.000 (2)	−0.001 (2)	0.017 (2)
C25_3	0.025 (3)	0.021 (2)	0.019 (2)	0.006 (2)	0.0054 (19)	0.007 (2)
C26_3	0.021 (2)	0.018 (2)	0.019 (2)	0.0037 (19)	0.0004 (18)	0.006 (2)
C27_3	0.032 (3)	0.031 (3)	0.042 (3)	0.015 (2)	0.014 (2)	0.017 (3)
C28_3	0.027 (3)	0.044 (3)	0.037 (3)	0.000 (2)	−0.001 (2)	0.028 (3)
C29_3	0.051 (3)	0.023 (3)	0.022 (3)	0.005 (2)	0.007 (2)	0.009 (2)
C30_3	0.057 (3)	0.025 (3)	0.022 (3)	0.002 (3)	0.010 (2)	0.008 (2)
C31_3	0.039 (3)	0.051 (3)	0.038 (3)	−0.003 (3)	−0.009 (2)	0.029 (3)
C32_3	0.041 (3)	0.040 (3)	0.056 (4)	0.027 (3)	0.018 (3)	0.036 (3)
C33_3	0.055 (3)	0.024 (3)	0.033 (3)	−0.002 (3)	0.007 (3)	0.012 (2)
C34_3	0.021 (2)	0.047 (3)	0.046 (3)	−0.004 (2)	−0.004 (2)	0.034 (3)
C35_3	0.048 (3)	0.049 (3)	0.045 (3)	0.024 (3)	0.026 (3)	0.035 (3)
O1_4	0.050 (2)	0.051 (2)	0.047 (2)	0.0199 (19)	0.0150 (18)	0.037 (2)
O2_4	0.0314 (18)	0.0162 (17)	0.0225 (18)	0.0079 (15)	−0.0013 (14)	0.0052 (15)
C1_4	0.032 (3)	0.036 (3)	0.040 (3)	0.000 (2)	0.000 (2)	0.028 (3)
C2_4	0.028 (3)	0.032 (3)	0.034 (3)	0.008 (2)	0.006 (2)	0.025 (2)
C3_4	0.020 (2)	0.018 (2)	0.023 (2)	0.0033 (19)	0.0030 (19)	0.008 (2)
C4_4	0.019 (2)	0.020 (2)	0.020 (2)	0.0053 (19)	0.0025 (18)	0.010 (2)
C5_4	0.036 (3)	0.033 (3)	0.027 (3)	0.018 (2)	0.008 (2)	0.019 (2)
C6_4	0.017 (2)	0.018 (2)	0.020 (2)	0.0046 (18)	0.0023 (17)	0.009 (2)
C7_4	0.024 (2)	0.021 (2)	0.026 (3)	0.009 (2)	0.0081 (19)	0.013 (2)
C8_4	0.029 (2)	0.018 (2)	0.020 (2)	0.011 (2)	0.0084 (19)	0.009 (2)
C9_4	0.018 (2)	0.015 (2)	0.020 (2)	0.0061 (18)	0.0026 (18)	0.0053 (19)
C10_4	0.016 (2)	0.013 (2)	0.021 (2)	0.0037 (18)	0.0021 (17)	0.0091 (19)
C11_4	0.022 (2)	0.021 (2)	0.024 (3)	0.0040 (19)	0.0023 (19)	0.013 (2)
C12_4	0.019 (2)	0.018 (2)	0.023 (2)	0.0077 (19)	0.0038 (18)	0.012 (2)
C13_4	0.024 (2)	0.023 (2)	0.022 (2)	0.013 (2)	0.0088 (19)	0.012 (2)
C14_4	0.019 (2)	0.016 (2)	0.021 (2)	0.0038 (18)	0.0016 (18)	0.010 (2)
C15_4	0.018 (2)	0.013 (2)	0.020 (2)	0.0015 (18)	0.0004 (17)	0.0073 (19)
C16_4	0.028 (2)	0.019 (2)	0.024 (3)	0.012 (2)	0.0078 (19)	0.011 (2)
C17_4	0.033 (3)	0.020 (2)	0.027 (3)	0.011 (2)	0.005 (2)	0.013 (2)
C18_4	0.021 (2)	0.020 (2)	0.027 (3)	0.0061 (19)	0.0054 (19)	0.015 (2)
C19_4	0.022 (2)	0.019 (2)	0.026 (3)	0.006 (2)	0.0066 (19)	0.012 (2)
C20_4	0.020 (2)	0.038 (3)	0.043 (3)	0.003 (2)	0.002 (2)	0.029 (3)
C21_4	0.043 (3)	0.031 (3)	0.030 (3)	0.021 (2)	0.018 (2)	0.024 (2)
C22_4	0.067 (4)	0.018 (2)	0.022 (3)	0.006 (3)	0.008 (2)	0.009 (2)
C23_4	0.036 (3)	0.025 (3)	0.029 (3)	−0.010 (2)	−0.006 (2)	0.017 (2)
C24_4	0.021 (2)	0.028 (3)	0.030 (3)	0.003 (2)	0.0005 (19)	0.019 (2)
N1_4	0.033 (2)	0.024 (2)	0.021 (2)	−0.0036 (18)	−0.0040 (17)	0.0126 (19)
C25_4	0.041 (6)	0.027 (4)	0.027 (5)	−0.007 (4)	−0.010 (4)	0.015 (4)
O10_4	0.110 (11)	0.046 (5)	0.045 (5)	−0.021 (5)	−0.021 (5)	0.027 (4)
C26_4	0.033 (4)	0.027 (3)	0.021 (4)	0.003 (3)	−0.003 (3)	0.013 (3)
C27_4	0.033 (4)	0.042 (4)	0.026 (4)	0.010 (4)	−0.001 (3)	0.019 (3)
C28_4	0.066 (5)	0.031 (4)	0.025 (4)	−0.001 (4)	0.006 (4)	0.012 (3)
C29_4	0.039 (4)	0.039 (4)	0.033 (4)	0.009 (4)	−0.004 (3)	0.019 (3)
C30_4	0.040 (4)	0.057 (4)	0.030 (5)	0.012 (4)	0.003 (4)	0.023 (4)
C31_4	0.035 (5)	0.044 (5)	0.027 (4)	0.012 (4)	0.008 (4)	0.016 (4)
C32_4	0.040 (4)	0.044 (4)	0.031 (4)	0.017 (4)	0.004 (3)	0.026 (3)
C33_4	0.072 (5)	0.046 (4)	0.039 (5)	0.026 (4)	0.020 (4)	0.028 (4)
C34_4	0.085 (5)	0.032 (4)	0.030 (4)	0.001 (4)	0.013 (4)	0.014 (4)
C35_4	0.068 (5)	0.054 (5)	0.036 (5)	−0.006 (5)	0.001 (4)	0.027 (4)
C25'_4	0.030 (5)	0.030 (5)	0.026 (5)	−0.003 (4)	0.004 (4)	0.008 (4)
O10'_4	0.091 (10)	0.086 (7)	0.051 (6)	−0.044 (7)	−0.005 (6)	0.012 (6)
C26'_4	0.030 (4)	0.030 (4)	0.022 (4)	0.001 (3)	0.003 (3)	0.008 (3)
C27'_4	0.052 (5)	0.045 (4)	0.039 (4)	0.018 (4)	0.015 (4)	0.018 (4)
C28'_4	0.036 (4)	0.036 (4)	0.028 (4)	−0.010 (4)	0.000 (4)	0.017 (4)
C29'_4	0.036 (4)	0.030 (4)	0.021 (4)	0.004 (4)	0.002 (3)	0.012 (3)
C30'_4	0.043 (4)	0.040 (4)	0.024 (4)	0.009 (4)	0.007 (3)	0.018 (3)
C31'_4	0.046 (5)	0.044 (5)	0.040 (4)	0.010 (4)	0.004 (4)	0.028 (4)
C32'_4	0.064 (5)	0.048 (5)	0.049 (5)	0.025 (4)	0.018 (4)	0.026 (4)
C33'_4	0.070 (5)	0.033 (5)	0.037 (5)	0.003 (4)	0.020 (4)	0.012 (4)
C34'_4	0.048 (4)	0.043 (5)	0.034 (4)	−0.004 (4)	0.001 (4)	0.025 (4)
C35'_4	0.043 (4)	0.050 (5)	0.032 (5)	0.014 (4)	0.010 (4)	0.028 (4)
O1W	0.053 (3)	0.050 (3)	0.060 (3)	−0.005 (2)	0.005 (2)	0.018 (2)

### Geometric parameters (Å, º)

**Table:**

O1_1—C1_1	1.427 (5)	C3_3—C4_3	1.521 (6)
O1_1—H1_1	0.88 (2)	C3_3—H3A_3	0.9900
O2_1—C12_1	1.431 (5)	C3_3—H3B_3	0.9900
O2_1—H2_1	0.87 (2)	C4_3—C6_3	1.535 (6)
C23_1—N1_1	1.484 (7)	C4_3—C5_3	1.538 (6)
C23_1—N1'_1	1.502 (13)	C4_3—H4_3	1.0000
C23_1—C24_1	1.508 (8)	C5_3—H5A_3	0.9800
C23_1—C22_1	1.528 (7)	C5_3—H5B_3	0.9800
C23_1—H23_1	1.0000	C5_3—H5C_3	0.9800
C25_1—O3_1	1.239 (8)	C6_3—C10_3	1.555 (6)
C25_1—N1_1	1.335 (9)	C6_3—C7_3	1.556 (6)
C25_1—C26_1	1.557 (8)	C6_3—H6_3	1.0000
N1_1—H1N_1	0.83 (3)	C7_3—C8_3	1.541 (6)
C26_1—C27_1	1.515 (6)	C7_3—H7A_3	0.9900
C26_1—C28_1	1.529 (7)	C7_3—H7B_3	0.9900
C26_1—C25'_1	1.531 (12)	C8_3—C9_3	1.527 (6)
C26_1—C29_1	1.538 (6)	C8_3—H8A_3	0.9900
C25'_1—O3'_1	1.233 (13)	C8_3—H8B_3	0.9900
C25'_1—N1'_1	1.326 (13)	C9_3—C15_3	1.525 (6)
N1'_1—H1N'_1	0.83 (3)	C9_3—C10_3	1.551 (6)
C1_1—C2_1	1.508 (6)	C9_3—H9_3	1.0000
C1_1—H1A_1	0.9900	C10_3—C11_3	1.526 (6)
C1_1—H1B_1	0.9900	C10_3—C12_3	1.538 (6)
C2_1—C3_1	1.526 (6)	C11_3—H11A_3	0.9800
C2_1—H2A_1	0.9900	C11_3—H11B_3	0.9800
C2_1—H2B_1	0.9900	C11_3—H11C_3	0.9800
C3_1—C4_1	1.541 (6)	C12_3—C13_3	1.532 (6)
C3_1—H3A_1	0.9900	C12_3—H12_3	1.0000
C3_1—H3B_1	0.9900	C13_3—C14_3	1.536 (6)
C4_1—C6_1	1.523 (6)	C13_3—H13A_3	0.9900
C4_1—C5_1	1.526 (6)	C13_3—H13B_3	0.9900
C4_1—H4_1	1.0000	C14_3—C15_3	1.543 (6)
C5_1—H5A_1	0.9800	C14_3—C19_3	1.554 (6)
C5_1—H5B_1	0.9800	C14_3—H14_3	1.0000
C5_1—H5C_1	0.9800	C15_3—C16_3	1.533 (6)
C6_1—C10_1	1.556 (6)	C15_3—H15_3	1.0000
C6_1—C7_1	1.559 (6)	C16_3—C17_3	1.530 (6)
C6_1—H6_1	1.0000	C16_3—H16A_3	0.9900
C7_1—C8_1	1.544 (6)	C16_3—H16B_3	0.9900
C7_1—H7A_1	0.9900	C17_3—C18_3	1.533 (6)
C7_1—H7B_1	0.9900	C17_3—H17A_3	0.9900
C8_1—C9_1	1.528 (6)	C17_3—H17B_3	0.9900
C8_1—H8A_1	0.9900	C18_3—C24_3	1.530 (6)
C8_1—H8B_1	0.9900	C18_3—C19_3	1.553 (6)
C9_1—C15_1	1.525 (6)	C18_3—H18_3	1.0000
C9_1—C10_1	1.535 (6)	C19_3—C20_3	1.535 (6)
C9_1—H9_1	1.0000	C19_3—C21_3	1.537 (6)
C10_1—C12_1	1.539 (6)	C20_3—H20A_3	0.9800
C10_1—C11_1	1.540 (6)	C20_3—H20B_3	0.9800
C11_1—H11A_1	0.9800	C20_3—H20C_3	0.9800
C11_1—H11B_1	0.9800	C21_3—C22_3	1.502 (7)
C11_1—H11C_1	0.9800	C21_3—H21A_3	0.9900
C12_1—C13_1	1.535 (6)	C21_3—H21B_3	0.9900
C12_1—H12_1	1.0000	C22_3—C23_3	1.529 (7)
C13_1—C14_1	1.538 (6)	C22_3—H22A_3	0.9900
C13_1—H13A_1	0.9900	C22_3—H22B_3	0.9900
C13_1—H13B_1	0.9900	C23_3—C24_3	1.534 (7)
C14_1—C15_1	1.546 (6)	C23_3—H23_3	1.0000
C14_1—C19_1	1.551 (6)	C24_3—H24A_3	0.9900
C14_1—H14_1	1.0000	C24_3—H24B_3	0.9900
C15_1—C16_1	1.514 (7)	C25_3—C26_3	1.524 (6)
C15_1—H15_1	1.0000	C26_3—C29_3	1.531 (6)
C16_1—C17_1	1.536 (7)	C26_3—C28_3	1.536 (6)
C16_1—H16A_1	0.9900	C26_3—C27_3	1.538 (6)
C16_1—H16B_1	0.9900	C27_3—C32_3	1.533 (7)
C17_1—C18_1	1.532 (7)	C27_3—H27A_3	0.9900
C17_1—H17A_1	0.9900	C27_3—H27B_3	0.9900
C17_1—H17B_1	0.9900	C28_3—C34_3	1.527 (7)
C18_1—C24_1	1.516 (7)	C28_3—H28A_3	0.9900
C18_1—C19_1	1.543 (6)	C28_3—H28B_3	0.9900
C18_1—H18_1	1.0000	C29_3—C30_3	1.530 (7)
C19_1—C21_1	1.536 (6)	C29_3—H29A_3	0.9900
C19_1—C20_1	1.544 (6)	C29_3—H29B_3	0.9900
C20_1—H20A_1	0.9800	C30_3—C31_3	1.512 (8)
C20_1—H20B_1	0.9800	C30_3—C35_3	1.522 (8)
C20_1—H20C_1	0.9800	C30_3—H30_3	1.0000
C21_1—C22_1	1.526 (6)	C31_3—C32_3	1.519 (7)
C21_1—H21A_1	0.9900	C31_3—H31A_3	0.9900
C21_1—H21B_1	0.9900	C31_3—H31B_3	0.9900
C22_1—H22A_1	0.9900	C32_3—C33_3	1.523 (7)
C22_1—H22B_1	0.9900	C32_3—H32_3	1.0000
C24_1—H24A_1	0.9900	C33_3—C34_3	1.516 (7)
C24_1—H24B_1	0.9900	C33_3—H33A_3	0.9900
C27_1—C32_1	1.541 (6)	C33_3—H33B_3	0.9900
C27_1—H27A_1	0.9900	C34_3—C35_3	1.503 (7)
C27_1—H27B_1	0.9900	C34_3—H34_3	1.0000
C28_1—C34_1	1.536 (7)	C35_3—H35A_3	0.9900
C28_1—H28A_1	0.9900	C35_3—H35B_3	0.9900
C28_1—H28B_1	0.9900	O1_4—C1_4	1.447 (6)
C29_1—C30_1	1.532 (7)	O1_4—H1_4	0.88 (2)
C29_1—H29A_1	0.9900	O2_4—C12_4	1.440 (5)
C29_1—H29B_1	0.9900	O2_4—H2_4	0.74 (5)
C30_1—C35_1	1.519 (6)	C1_4—C2_4	1.501 (6)
C30_1—C31_1	1.527 (7)	C1_4—H1A_4	0.9900
C30_1—H30_1	1.0000	C1_4—H1B_4	0.9900
C31_1—C32_1	1.535 (7)	C2_4—C3_4	1.536 (6)
C31_1—H31A_1	0.9900	C2_4—H2A_4	0.9900
C31_1—H31B_1	0.9900	C2_4—H2B_4	0.9900
C32_1—C33_1	1.537 (7)	C3_4—C4_4	1.541 (6)
C32_1—H32_1	1.0000	C3_4—H3A_4	0.9900
C33_1—C34_1	1.507 (7)	C3_4—H3B_4	0.9900
C33_1—H33A_1	0.9900	C4_4—C5_4	1.529 (6)
C33_1—H33B_1	0.9900	C4_4—C6_4	1.543 (6)
C34_1—C35_1	1.528 (6)	C4_4—H4_4	1.0000
C34_1—H34_1	1.0000	C5_4—H5A_4	0.9800
C35_1—H35A_1	0.9900	C5_4—H5B_4	0.9800
C35_1—H35B_1	0.9900	C5_4—H5C_4	0.9800
O1_2—C1_2	1.402 (6)	C6_4—C10_4	1.549 (6)
O1_2—H1_2	0.88 (2)	C6_4—C7_4	1.551 (6)
O2_2—C12_2	1.431 (5)	C6_4—H6_4	1.0000
O2_2—H2_2	0.87 (2)	C7_4—C8_4	1.554 (6)
O3_2—C25_2	1.216 (6)	C7_4—H7A_4	0.9900
N1_2—C25_2	1.329 (6)	C7_4—H7B_4	0.9900
N1_2—C23_2	1.473 (6)	C8_4—C9_4	1.532 (6)
N1_2—H1N_2	0.83 (3)	C8_4—H8A_4	0.9900
C1_2—C2_2	1.524 (7)	C8_4—H8B_4	0.9900
C1_2—H1A_2	0.9900	C9_4—C15_4	1.523 (6)
C1_2—H1B_2	0.9900	C9_4—C10_4	1.546 (6)
C2_2—C3_2	1.514 (7)	C9_4—H9_4	1.0000
C2_2—H2A_2	0.9900	C10_4—C12_4	1.531 (6)
C2_2—H2B_2	0.9900	C10_4—C11_4	1.536 (6)
C3_2—C4_2	1.526 (6)	C11_4—H11A_4	0.9800
C3_2—H3A_2	0.9900	C11_4—H11B_4	0.9800
C3_2—H3B_2	0.9900	C11_4—H11C_4	0.9800
C4_2—C5_2	1.531 (6)	C12_4—C13_4	1.529 (6)
C4_2—C6_2	1.536 (6)	C12_4—H12_4	1.0000
C4_2—H4_2	1.0000	C13_4—C14_4	1.539 (6)
C5_2—H5A_2	0.9800	C13_4—H13A_4	0.9900
C5_2—H5B_2	0.9800	C13_4—H13B_4	0.9900
C5_2—H5C_2	0.9800	C14_4—C15_4	1.536 (6)
C6_2—C10_2	1.559 (6)	C14_4—C19_4	1.556 (6)
C6_2—C7_2	1.563 (6)	C14_4—H14_4	1.0000
C6_2—H6_2	1.0000	C15_4—C16_4	1.529 (6)
C7_2—C8_2	1.536 (7)	C15_4—H15_4	1.0000
C7_2—H7A_2	0.9900	C16_4—C17_4	1.528 (6)
C7_2—H7B_2	0.9900	C16_4—H16A_4	0.9900
C8_2—C9_2	1.528 (6)	C16_4—H16B_4	0.9900
C8_2—H8A_2	0.9900	C17_4—C18_4	1.528 (6)
C8_2—H8B_2	0.9900	C17_4—H17A_4	0.9900
C9_2—C15_2	1.516 (6)	C17_4—H17B_4	0.9900
C9_2—C10_2	1.538 (6)	C18_4—C24_4	1.530 (6)
C9_2—H9_2	1.0000	C18_4—C19_4	1.545 (6)
C10_2—C12_2	1.536 (6)	C18_4—H18_4	1.0000
C10_2—C11_2	1.546 (6)	C19_4—C21_4	1.531 (6)
C11_2—H11A_2	0.9800	C19_4—C20_4	1.543 (6)
C11_2—H11B_2	0.9800	C20_4—H20A_4	0.9800
C11_2—H11C_2	0.9800	C20_4—H20B_4	0.9800
C12_2—C13_2	1.538 (6)	C20_4—H20C_4	0.9800
C12_2—H12_2	1.0000	C21_4—C22_4	1.525 (7)
C13_2—C14_2	1.537 (6)	C21_4—H21A_4	0.9900
C13_2—H13A_2	0.9900	C21_4—H21B_4	0.9900
C13_2—H13B_2	0.9900	C22_4—C23_4	1.520 (7)
C14_2—C15_2	1.543 (6)	C22_4—H22A_4	0.9900
C14_2—C19_2	1.556 (6)	C22_4—H22B_4	0.9900
C14_2—H14_2	1.0000	C23_4—N1_4	1.460 (6)
C15_2—C16_2	1.537 (6)	C23_4—C24_4	1.518 (7)
C15_2—H15_2	1.0000	C23_4—H23_4	1.0000
C16_2—C17_2	1.520 (7)	C24_4—H24A_4	0.9900
C16_2—H16A_2	0.9900	C24_4—H24B_4	0.9900
C16_2—H16B_2	0.9900	N1_4—C25'_4	1.367 (9)
C17_2—C18_2	1.520 (7)	N1_4—C25_4	1.370 (9)
C17_2—H17A_2	0.9900	N1_4—H1N_4	0.83 (3)
C17_2—H17B_2	0.9900	C25_4—O10_4	1.224 (9)
C18_2—C24_2	1.534 (6)	C25_4—C26_4	1.531 (8)
C18_2—C19_2	1.561 (6)	C26_4—C27_4	1.525 (9)
C18_2—H18_2	1.0000	C26_4—C29_4	1.529 (9)
C19_2—C21_2	1.531 (7)	C26_4—C28_4	1.537 (9)
C19_2—C20_2	1.539 (6)	C27_4—C32_4	1.518 (10)
C20_2—H20A_2	0.9800	C27_4—H27A_4	0.9900
C20_2—H20B_2	0.9800	C27_4—H27B_4	0.9900
C20_2—H20C_2	0.9800	C28_4—C34_4	1.542 (9)
C21_2—C22_2	1.530 (7)	C28_4—H28A_4	0.9900
C21_2—H21A_2	0.9900	C28_4—H28B_4	0.9900
C21_2—H21B_2	0.9900	C29_4—C30_4	1.526 (10)
C22_2—C23_2	1.517 (7)	C29_4—H29A_4	0.9900
C22_2—H22A_2	0.9900	C29_4—H29B_4	0.9900
C22_2—H22B_2	0.9900	C30_4—C35_4	1.512 (10)
C23_2—C24_2	1.515 (7)	C30_4—C31_4	1.536 (9)
C23_2—H23_2	1.0000	C30_4—H30_4	1.0000
C24_2—H24A_2	0.9900	C31_4—C32_4	1.529 (9)
C24_2—H24B_2	0.9900	C31_4—H31A_4	0.9900
C25_2—C26_2	1.519 (7)	C31_4—H31B_4	0.9900
C26_2—C28_2	1.527 (6)	C32_4—C33_4	1.532 (10)
C26_2—C27_2	1.528 (7)	C32_4—H32_4	1.0000
C26_2—C29_2	1.548 (7)	C33_4—C34_4	1.522 (10)
C27_2—C32_2	1.546 (7)	C33_4—H33A_4	0.9900
C27_2—H27A_2	0.9900	C33_4—H33B_4	0.9900
C27_2—H27B_2	0.9900	C34_4—C35_4	1.520 (11)
C28_2—C34_2	1.531 (7)	C34_4—H34_4	1.0000
C28_2—H28A_2	0.9900	C35_4—H35A_4	0.9900
C28_2—H28B_2	0.9900	C35_4—H35B_4	0.9900
C29_2—C30_2	1.534 (7)	C25'_4—O10'_4	1.220 (10)
C29_2—H29A_2	0.9900	C25'_4—C26'_4	1.531 (9)
C29_2—H29B_2	0.9900	C26'_4—C29'_4	1.523 (9)
C30_2—C31_2	1.516 (7)	C26'_4—C27'_4	1.524 (10)
C30_2—C35_2	1.519 (7)	C26'_4—C28'_4	1.542 (9)
C30_2—H30_2	1.0000	C27'_4—C32'_4	1.520 (10)
C31_2—C32_2	1.525 (8)	C27'_4—H27C_4	0.9900
C31_2—H31A_2	0.9900	C27'_4—H27D_4	0.9900
C31_2—H31B_2	0.9900	C28'_4—C34'_4	1.537 (9)
C32_2—C33_2	1.526 (8)	C28'_4—H28C_4	0.9900
C32_2—H32_2	1.0000	C28'_4—H28D_4	0.9900
C33_2—C34_2	1.508 (8)	C29'_4—C30'_4	1.529 (9)
C33_2—H33A_2	0.9900	C29'_4—H29C_4	0.9900
C33_2—H33B_2	0.9900	C29'_4—H29D_4	0.9900
C34_2—C35_2	1.523 (7)	C30'_4—C35'_4	1.513 (10)
C34_2—H34_2	1.0000	C30'_4—C31'_4	1.529 (9)
C35_2—H35A_2	0.9900	C30'_4—H30'_4	1.0000
C35_2—H35B_2	0.9900	C31'_4—C32'_4	1.547 (10)
O1_3—C1_3	1.416 (6)	C31'_4—H31C_4	0.9900
O1_3—H1_3	0.88 (2)	C31'_4—H31D_4	0.9900
O2_3—C12_3	1.437 (5)	C32'_4—C33'_4	1.532 (11)
O2_3—H2_3	0.87 (2)	C32'_4—H32'_4	1.0000
O3_3—C25_3	1.235 (5)	C33'_4—C34'_4	1.510 (11)
N1_3—C25_3	1.336 (6)	C33'_4—H33C_4	0.9900
N1_3—C23_3	1.473 (6)	C33'_4—H33D_4	0.9900
N1_3—H1N_3	0.83 (3)	C34'_4—C35'_4	1.519 (10)
C1_3—C2_3	1.503 (6)	C34'_4—H34'_4	1.0000
C1_3—H1A_3	0.9900	C35'_4—H35C_4	0.9900
C1_3—H1B_3	0.9900	C35'_4—H35D_4	0.9900
C2_3—C3_3	1.527 (6)	O1W—H1W	0.855 (15)
C2_3—H2A_3	0.9900	O1W—H2W	0.858 (15)
C2_3—H2B_3	0.9900		
			
C1_1—O1_1—H1_1	106 (4)	C5_3—C4_3—H4_3	108.1
C12_1—O2_1—H2_1	111 (3)	C4_3—C5_3—H5A_3	109.5
N1_1—C23_1—C24_1	107.3 (5)	C4_3—C5_3—H5B_3	109.5
N1'_1—C23_1—C24_1	121.7 (11)	H5A_3—C5_3—H5B_3	109.5
N1_1—C23_1—C22_1	113.3 (5)	C4_3—C5_3—H5C_3	109.5
N1'_1—C23_1—C22_1	99.1 (9)	H5A_3—C5_3—H5C_3	109.5
C24_1—C23_1—C22_1	112.6 (4)	H5B_3—C5_3—H5C_3	109.5
N1'_1—C23_1—H23_1	107.5	C4_3—C6_3—C10_3	118.8 (3)
C24_1—C23_1—H23_1	107.5	C4_3—C6_3—C7_3	113.4 (3)
C22_1—C23_1—H23_1	107.5	C10_3—C6_3—C7_3	103.2 (3)
O3_1—C25_1—N1_1	120.7 (6)	C4_3—C6_3—H6_3	106.9
O3_1—C25_1—C26_1	124.4 (6)	C10_3—C6_3—H6_3	106.9
N1_1—C25_1—C26_1	114.7 (6)	C7_3—C6_3—H6_3	106.9
C25_1—N1_1—C23_1	119.9 (6)	C8_3—C7_3—C6_3	106.7 (3)
C25_1—N1_1—H1N_1	120 (4)	C8_3—C7_3—H7A_3	110.4
C23_1—N1_1—H1N_1	119 (4)	C6_3—C7_3—H7A_3	110.4
C27_1—C26_1—C28_1	109.0 (4)	C8_3—C7_3—H7B_3	110.4
C27_1—C26_1—C25'_1	112.7 (9)	C6_3—C7_3—H7B_3	110.4
C28_1—C26_1—C25'_1	97.8 (11)	H7A_3—C7_3—H7B_3	108.6
C27_1—C26_1—C29_1	108.8 (4)	C9_3—C8_3—C7_3	105.0 (3)
C28_1—C26_1—C29_1	109.1 (4)	C9_3—C8_3—H8A_3	110.7
C25'_1—C26_1—C29_1	118.6 (7)	C7_3—C8_3—H8A_3	110.7
C27_1—C26_1—C25_1	106.6 (4)	C9_3—C8_3—H8B_3	110.7
C28_1—C26_1—C25_1	113.2 (4)	C7_3—C8_3—H8B_3	110.7
C29_1—C26_1—C25_1	110.1 (4)	H8A_3—C8_3—H8B_3	108.8
O3'_1—C25'_1—N1'_1	123.2 (12)	C15_3—C9_3—C8_3	119.1 (3)
O3'_1—C25'_1—C26_1	117.1 (11)	C15_3—C9_3—C10_3	113.8 (3)
N1'_1—C25'_1—C26_1	119.7 (11)	C8_3—C9_3—C10_3	104.2 (3)
C25'_1—N1'_1—C23_1	116.7 (13)	C15_3—C9_3—H9_3	106.3
C25'_1—N1'_1—H1N'_1	124 (6)	C8_3—C9_3—H9_3	106.3
C23_1—N1'_1—H1N'_1	119 (5)	C10_3—C9_3—H9_3	106.3
O1_1—C1_1—C2_1	113.8 (4)	C11_3—C10_3—C12_3	109.5 (3)
O1_1—C1_1—H1A_1	108.8	C11_3—C10_3—C9_3	113.3 (3)
C2_1—C1_1—H1A_1	108.8	C12_3—C10_3—C9_3	107.1 (3)
O1_1—C1_1—H1B_1	108.8	C11_3—C10_3—C6_3	109.8 (3)
C2_1—C1_1—H1B_1	108.8	C12_3—C10_3—C6_3	116.6 (3)
H1A_1—C1_1—H1B_1	107.7	C9_3—C10_3—C6_3	100.5 (3)
C1_1—C2_1—C3_1	112.1 (4)	C10_3—C11_3—H11A_3	109.5
C1_1—C2_1—H2A_1	109.2	C10_3—C11_3—H11B_3	109.5
C3_1—C2_1—H2A_1	109.2	H11A_3—C11_3—H11B_3	109.5
C1_1—C2_1—H2B_1	109.2	C10_3—C11_3—H11C_3	109.5
C3_1—C2_1—H2B_1	109.2	H11A_3—C11_3—H11C_3	109.5
H2A_1—C2_1—H2B_1	107.9	H11B_3—C11_3—H11C_3	109.5
C2_1—C3_1—C4_1	114.8 (4)	O2_3—C12_3—C13_3	109.0 (3)
C2_1—C3_1—H3A_1	108.6	O2_3—C12_3—C10_3	110.3 (3)
C4_1—C3_1—H3A_1	108.6	C13_3—C12_3—C10_3	110.6 (3)
C2_1—C3_1—H3B_1	108.6	O2_3—C12_3—H12_3	109.0
C4_1—C3_1—H3B_1	108.6	C13_3—C12_3—H12_3	109.0
H3A_1—C3_1—H3B_1	107.5	C10_3—C12_3—H12_3	109.0
C6_1—C4_1—C5_1	113.4 (4)	C12_3—C13_3—C14_3	113.9 (3)
C6_1—C4_1—C3_1	110.7 (4)	C12_3—C13_3—H13A_3	108.8
C5_1—C4_1—C3_1	109.7 (4)	C14_3—C13_3—H13A_3	108.8
C6_1—C4_1—H4_1	107.6	C12_3—C13_3—H13B_3	108.8
C5_1—C4_1—H4_1	107.6	C14_3—C13_3—H13B_3	108.8
C3_1—C4_1—H4_1	107.6	H13A_3—C13_3—H13B_3	107.7
C4_1—C5_1—H5A_1	109.5	C13_3—C14_3—C15_3	112.1 (3)
C4_1—C5_1—H5B_1	109.5	C13_3—C14_3—C19_3	113.4 (3)
H5A_1—C5_1—H5B_1	109.5	C15_3—C14_3—C19_3	112.5 (3)
C4_1—C5_1—H5C_1	109.5	C13_3—C14_3—H14_3	106.0
H5A_1—C5_1—H5C_1	109.5	C15_3—C14_3—H14_3	106.0
H5B_1—C5_1—H5C_1	109.5	C19_3—C14_3—H14_3	106.0
C4_1—C6_1—C10_1	119.0 (4)	C9_3—C15_3—C16_3	111.7 (3)
C4_1—C6_1—C7_1	112.3 (4)	C9_3—C15_3—C14_3	108.2 (3)
C10_1—C6_1—C7_1	102.6 (3)	C16_3—C15_3—C14_3	110.2 (3)
C4_1—C6_1—H6_1	107.4	C9_3—C15_3—H15_3	108.9
C10_1—C6_1—H6_1	107.4	C16_3—C15_3—H15_3	108.9
C7_1—C6_1—H6_1	107.4	C14_3—C15_3—H15_3	108.9
C8_1—C7_1—C6_1	107.7 (4)	C17_3—C16_3—C15_3	112.4 (4)
C8_1—C7_1—H7A_1	110.2	C17_3—C16_3—H16A_3	109.1
C6_1—C7_1—H7A_1	110.2	C15_3—C16_3—H16A_3	109.1
C8_1—C7_1—H7B_1	110.2	C17_3—C16_3—H16B_3	109.1
C6_1—C7_1—H7B_1	110.2	C15_3—C16_3—H16B_3	109.1
H7A_1—C7_1—H7B_1	108.5	H16A_3—C16_3—H16B_3	107.9
C9_1—C8_1—C7_1	103.3 (4)	C16_3—C17_3—C18_3	112.7 (4)
C9_1—C8_1—H8A_1	111.1	C16_3—C17_3—H17A_3	109.1
C7_1—C8_1—H8A_1	111.1	C18_3—C17_3—H17A_3	109.1
C9_1—C8_1—H8B_1	111.1	C16_3—C17_3—H17B_3	109.1
C7_1—C8_1—H8B_1	111.1	C18_3—C17_3—H17B_3	109.1
H8A_1—C8_1—H8B_1	109.1	H17A_3—C17_3—H17B_3	107.8
C15_1—C9_1—C8_1	118.3 (4)	C24_3—C18_3—C17_3	110.3 (4)
C15_1—C9_1—C10_1	114.4 (4)	C24_3—C18_3—C19_3	112.6 (4)
C8_1—C9_1—C10_1	104.4 (4)	C17_3—C18_3—C19_3	111.9 (4)
C15_1—C9_1—H9_1	106.3	C24_3—C18_3—H18_3	107.2
C8_1—C9_1—H9_1	106.3	C17_3—C18_3—H18_3	107.2
C10_1—C9_1—H9_1	106.3	C19_3—C18_3—H18_3	107.2
C9_1—C10_1—C12_1	108.8 (3)	C20_3—C19_3—C21_3	107.2 (4)
C9_1—C10_1—C11_1	112.3 (4)	C20_3—C19_3—C18_3	109.6 (4)
C12_1—C10_1—C11_1	107.7 (3)	C21_3—C19_3—C18_3	107.9 (4)
C9_1—C10_1—C6_1	100.8 (3)	C20_3—C19_3—C14_3	111.2 (4)
C12_1—C10_1—C6_1	118.4 (3)	C21_3—C19_3—C14_3	111.6 (4)
C11_1—C10_1—C6_1	108.8 (3)	C18_3—C19_3—C14_3	109.3 (3)
C10_1—C11_1—H11A_1	109.5	C19_3—C20_3—H20A_3	109.5
C10_1—C11_1—H11B_1	109.5	C19_3—C20_3—H20B_3	109.5
H11A_1—C11_1—H11B_1	109.5	H20A_3—C20_3—H20B_3	109.5
C10_1—C11_1—H11C_1	109.5	C19_3—C20_3—H20C_3	109.5
H11A_1—C11_1—H11C_1	109.5	H20A_3—C20_3—H20C_3	109.5
H11B_1—C11_1—H11C_1	109.5	H20B_3—C20_3—H20C_3	109.5
O2_1—C12_1—C13_1	110.1 (3)	C22_3—C21_3—C19_3	114.5 (4)
O2_1—C12_1—C10_1	109.8 (3)	C22_3—C21_3—H21A_3	108.6
C13_1—C12_1—C10_1	111.6 (3)	C19_3—C21_3—H21A_3	108.6
O2_1—C12_1—H12_1	108.5	C22_3—C21_3—H21B_3	108.6
C13_1—C12_1—H12_1	108.5	C19_3—C21_3—H21B_3	108.6
C10_1—C12_1—H12_1	108.5	H21A_3—C21_3—H21B_3	107.6
C12_1—C13_1—C14_1	114.1 (3)	C21_3—C22_3—C23_3	111.5 (4)
C12_1—C13_1—H13A_1	108.7	C21_3—C22_3—H22A_3	109.3
C14_1—C13_1—H13A_1	108.7	C23_3—C22_3—H22A_3	109.3
C12_1—C13_1—H13B_1	108.7	C21_3—C22_3—H22B_3	109.3
C14_1—C13_1—H13B_1	108.7	C23_3—C22_3—H22B_3	109.3
H13A_1—C13_1—H13B_1	107.6	H22A_3—C22_3—H22B_3	108.0
C13_1—C14_1—C15_1	109.5 (3)	N1_3—C23_3—C22_3	108.4 (4)
C13_1—C14_1—C19_1	114.8 (3)	N1_3—C23_3—C24_3	112.7 (4)
C15_1—C14_1—C19_1	112.4 (3)	C22_3—C23_3—C24_3	110.8 (4)
C13_1—C14_1—H14_1	106.5	N1_3—C23_3—H23_3	108.3
C15_1—C14_1—H14_1	106.5	C22_3—C23_3—H23_3	108.3
C19_1—C14_1—H14_1	106.5	C24_3—C23_3—H23_3	108.3
C16_1—C15_1—C9_1	110.9 (4)	C18_3—C24_3—C23_3	115.9 (4)
C16_1—C15_1—C14_1	112.8 (4)	C18_3—C24_3—H24A_3	108.3
C9_1—C15_1—C14_1	108.5 (3)	C23_3—C24_3—H24A_3	108.3
C16_1—C15_1—H15_1	108.2	C18_3—C24_3—H24B_3	108.3
C9_1—C15_1—H15_1	108.2	C23_3—C24_3—H24B_3	108.3
C14_1—C15_1—H15_1	108.2	H24A_3—C24_3—H24B_3	107.4
C15_1—C16_1—C17_1	112.6 (4)	O3_3—C25_3—N1_3	121.7 (4)
C15_1—C16_1—H16A_1	109.1	O3_3—C25_3—C26_3	120.8 (4)
C17_1—C16_1—H16A_1	109.1	N1_3—C25_3—C26_3	117.5 (4)
C15_1—C16_1—H16B_1	109.1	C25_3—C26_3—C29_3	107.4 (3)
C17_1—C16_1—H16B_1	109.1	C25_3—C26_3—C28_3	115.4 (4)
H16A_1—C16_1—H16B_1	107.8	C29_3—C26_3—C28_3	108.2 (4)
C18_1—C17_1—C16_1	111.0 (4)	C25_3—C26_3—C27_3	109.0 (4)
C18_1—C17_1—H17A_1	109.4	C29_3—C26_3—C27_3	108.3 (4)
C16_1—C17_1—H17A_1	109.4	C28_3—C26_3—C27_3	108.4 (4)
C18_1—C17_1—H17B_1	109.4	C32_3—C27_3—C26_3	110.3 (4)
C16_1—C17_1—H17B_1	109.4	C32_3—C27_3—H27A_3	109.6
H17A_1—C17_1—H17B_1	108.0	C26_3—C27_3—H27A_3	109.6
C24_1—C18_1—C17_1	110.9 (4)	C32_3—C27_3—H27B_3	109.6
C24_1—C18_1—C19_1	112.3 (4)	C26_3—C27_3—H27B_3	109.6
C17_1—C18_1—C19_1	111.5 (4)	H27A_3—C27_3—H27B_3	108.1
C24_1—C18_1—H18_1	107.3	C34_3—C28_3—C26_3	110.0 (4)
C17_1—C18_1—H18_1	107.3	C34_3—C28_3—H28A_3	109.7
C19_1—C18_1—H18_1	107.3	C26_3—C28_3—H28A_3	109.7
C21_1—C19_1—C18_1	107.5 (4)	C34_3—C28_3—H28B_3	109.7
C21_1—C19_1—C20_1	106.1 (4)	C26_3—C28_3—H28B_3	109.7
C18_1—C19_1—C20_1	110.3 (4)	H28A_3—C28_3—H28B_3	108.2
C21_1—C19_1—C14_1	112.9 (3)	C30_3—C29_3—C26_3	110.7 (4)
C18_1—C19_1—C14_1	108.6 (3)	C30_3—C29_3—H29A_3	109.5
C20_1—C19_1—C14_1	111.3 (4)	C26_3—C29_3—H29A_3	109.5
C19_1—C20_1—H20A_1	109.5	C30_3—C29_3—H29B_3	109.5
C19_1—C20_1—H20B_1	109.5	C26_3—C29_3—H29B_3	109.5
H20A_1—C20_1—H20B_1	109.5	H29A_3—C29_3—H29B_3	108.1
C19_1—C20_1—H20C_1	109.5	C31_3—C30_3—C35_3	109.4 (4)
H20A_1—C20_1—H20C_1	109.5	C31_3—C30_3—C29_3	109.5 (4)
H20B_1—C20_1—H20C_1	109.5	C35_3—C30_3—C29_3	109.0 (4)
C22_1—C21_1—C19_1	113.5 (4)	C31_3—C30_3—H30_3	109.6
C22_1—C21_1—H21A_1	108.9	C35_3—C30_3—H30_3	109.6
C19_1—C21_1—H21A_1	108.9	C29_3—C30_3—H30_3	109.6
C22_1—C21_1—H21B_1	108.9	C30_3—C31_3—C32_3	109.9 (4)
C19_1—C21_1—H21B_1	108.9	C30_3—C31_3—H31A_3	109.7
H21A_1—C21_1—H21B_1	107.7	C32_3—C31_3—H31A_3	109.7
C21_1—C22_1—C23_1	112.4 (4)	C30_3—C31_3—H31B_3	109.7
C21_1—C22_1—H22A_1	109.1	C32_3—C31_3—H31B_3	109.7
C23_1—C22_1—H22A_1	109.1	H31A_3—C31_3—H31B_3	108.2
C21_1—C22_1—H22B_1	109.1	C31_3—C32_3—C33_3	109.6 (4)
C23_1—C22_1—H22B_1	109.1	C31_3—C32_3—C27_3	109.3 (4)
H22A_1—C22_1—H22B_1	107.9	C33_3—C32_3—C27_3	109.4 (4)
C23_1—C24_1—C18_1	115.0 (4)	C31_3—C32_3—H32_3	109.5
C23_1—C24_1—H24A_1	108.5	C33_3—C32_3—H32_3	109.5
C18_1—C24_1—H24A_1	108.5	C27_3—C32_3—H32_3	109.5
C23_1—C24_1—H24B_1	108.5	C34_3—C33_3—C32_3	108.9 (4)
C18_1—C24_1—H24B_1	108.5	C34_3—C33_3—H33A_3	109.9
H24A_1—C24_1—H24B_1	107.5	C32_3—C33_3—H33A_3	109.9
C26_1—C27_1—C32_1	110.5 (4)	C34_3—C33_3—H33B_3	109.9
C26_1—C27_1—H27A_1	109.6	C32_3—C33_3—H33B_3	109.9
C32_1—C27_1—H27A_1	109.6	H33A_3—C33_3—H33B_3	108.3
C26_1—C27_1—H27B_1	109.6	C35_3—C34_3—C33_3	110.4 (4)
C32_1—C27_1—H27B_1	109.6	C35_3—C34_3—C28_3	109.8 (4)
H27A_1—C27_1—H27B_1	108.1	C33_3—C34_3—C28_3	109.7 (4)
C26_1—C28_1—C34_1	109.8 (4)	C35_3—C34_3—H34_3	108.9
C26_1—C28_1—H28A_1	109.7	C33_3—C34_3—H34_3	108.9
C34_1—C28_1—H28A_1	109.7	C28_3—C34_3—H34_3	108.9
C26_1—C28_1—H28B_1	109.7	C34_3—C35_3—C30_3	109.4 (4)
C34_1—C28_1—H28B_1	109.7	C34_3—C35_3—H35A_3	109.8
H28A_1—C28_1—H28B_1	108.2	C30_3—C35_3—H35A_3	109.8
C30_1—C29_1—C26_1	110.0 (4)	C34_3—C35_3—H35B_3	109.8
C30_1—C29_1—H29A_1	109.7	C30_3—C35_3—H35B_3	109.8
C26_1—C29_1—H29A_1	109.7	H35A_3—C35_3—H35B_3	108.2
C30_1—C29_1—H29B_1	109.7	C1_4—O1_4—H1_4	109 (4)
C26_1—C29_1—H29B_1	109.7	C12_4—O2_4—H2_4	106 (4)
H29A_1—C29_1—H29B_1	108.2	O1_4—C1_4—C2_4	112.2 (4)
C35_1—C30_1—C31_1	109.6 (4)	O1_4—C1_4—H1A_4	109.2
C35_1—C30_1—C29_1	110.1 (4)	C2_4—C1_4—H1A_4	109.2
C31_1—C30_1—C29_1	108.9 (4)	O1_4—C1_4—H1B_4	109.2
C35_1—C30_1—H30_1	109.4	C2_4—C1_4—H1B_4	109.2
C31_1—C30_1—H30_1	109.4	H1A_4—C1_4—H1B_4	107.9
C29_1—C30_1—H30_1	109.4	C1_4—C2_4—C3_4	113.0 (4)
C30_1—C31_1—C32_1	109.6 (4)	C1_4—C2_4—H2A_4	109.0
C30_1—C31_1—H31A_1	109.7	C3_4—C2_4—H2A_4	109.0
C32_1—C31_1—H31A_1	109.7	C1_4—C2_4—H2B_4	109.0
C30_1—C31_1—H31B_1	109.7	C3_4—C2_4—H2B_4	109.0
C32_1—C31_1—H31B_1	109.7	H2A_4—C2_4—H2B_4	107.8
H31A_1—C31_1—H31B_1	108.2	C2_4—C3_4—C4_4	113.3 (3)
C31_1—C32_1—C33_1	108.9 (4)	C2_4—C3_4—H3A_4	108.9
C31_1—C32_1—C27_1	108.9 (4)	C4_4—C3_4—H3A_4	108.9
C33_1—C32_1—C27_1	109.4 (4)	C2_4—C3_4—H3B_4	108.9
C31_1—C32_1—H32_1	109.9	C4_4—C3_4—H3B_4	108.9
C33_1—C32_1—H32_1	109.9	H3A_4—C3_4—H3B_4	107.7
C27_1—C32_1—H32_1	109.9	C5_4—C4_4—C3_4	108.9 (4)
C34_1—C33_1—C32_1	109.5 (4)	C5_4—C4_4—C6_4	111.0 (3)
C34_1—C33_1—H33A_1	109.8	C3_4—C4_4—C6_4	112.1 (3)
C32_1—C33_1—H33A_1	109.8	C5_4—C4_4—H4_4	108.3
C34_1—C33_1—H33B_1	109.8	C3_4—C4_4—H4_4	108.3
C32_1—C33_1—H33B_1	109.8	C6_4—C4_4—H4_4	108.3
H33A_1—C33_1—H33B_1	108.2	C4_4—C5_4—H5A_4	109.5
C33_1—C34_1—C35_1	109.8 (4)	C4_4—C5_4—H5B_4	109.5
C33_1—C34_1—C28_1	110.0 (4)	H5A_4—C5_4—H5B_4	109.5
C35_1—C34_1—C28_1	109.5 (4)	C4_4—C5_4—H5C_4	109.5
C33_1—C34_1—H34_1	109.2	H5A_4—C5_4—H5C_4	109.5
C35_1—C34_1—H34_1	109.2	H5B_4—C5_4—H5C_4	109.5
C28_1—C34_1—H34_1	109.2	C4_4—C6_4—C10_4	118.4 (3)
C30_1—C35_1—C34_1	109.3 (4)	C4_4—C6_4—C7_4	113.1 (3)
C30_1—C35_1—H35A_1	109.8	C10_4—C6_4—C7_4	103.2 (3)
C34_1—C35_1—H35A_1	109.8	C4_4—C6_4—H6_4	107.2
C30_1—C35_1—H35B_1	109.8	C10_4—C6_4—H6_4	107.2
C34_1—C35_1—H35B_1	109.8	C7_4—C6_4—H6_4	107.2
H35A_1—C35_1—H35B_1	108.3	C6_4—C7_4—C8_4	106.7 (3)
C1_2—O1_2—H1_2	106 (5)	C6_4—C7_4—H7A_4	110.4
C12_2—O2_2—H2_2	103 (3)	C8_4—C7_4—H7A_4	110.4
C25_2—N1_2—C23_2	121.9 (4)	C6_4—C7_4—H7B_4	110.4
C25_2—N1_2—H1N_2	119 (4)	C8_4—C7_4—H7B_4	110.4
C23_2—N1_2—H1N_2	119 (4)	H7A_4—C7_4—H7B_4	108.6
O1_2—C1_2—C2_2	109.6 (5)	C9_4—C8_4—C7_4	104.3 (3)
O1_2—C1_2—H1A_2	109.7	C9_4—C8_4—H8A_4	110.9
C2_2—C1_2—H1A_2	109.7	C7_4—C8_4—H8A_4	110.9
O1_2—C1_2—H1B_2	109.7	C9_4—C8_4—H8B_4	110.9
C2_2—C1_2—H1B_2	109.7	C7_4—C8_4—H8B_4	110.9
H1A_2—C1_2—H1B_2	108.2	H8A_4—C8_4—H8B_4	108.9
C3_2—C2_2—C1_2	111.1 (4)	C15_4—C9_4—C8_4	117.9 (3)
C3_2—C2_2—H2A_2	109.4	C15_4—C9_4—C10_4	114.8 (3)
C1_2—C2_2—H2A_2	109.4	C8_4—C9_4—C10_4	104.1 (3)
C3_2—C2_2—H2B_2	109.4	C15_4—C9_4—H9_4	106.4
C1_2—C2_2—H2B_2	109.4	C8_4—C9_4—H9_4	106.4
H2A_2—C2_2—H2B_2	108.0	C10_4—C9_4—H9_4	106.4
C2_2—C3_2—C4_2	114.6 (4)	C12_4—C10_4—C11_4	107.9 (3)
C2_2—C3_2—H3A_2	108.6	C12_4—C10_4—C9_4	107.3 (3)
C4_2—C3_2—H3A_2	108.6	C11_4—C10_4—C9_4	112.2 (3)
C2_2—C3_2—H3B_2	108.6	C12_4—C10_4—C6_4	119.1 (3)
C4_2—C3_2—H3B_2	108.6	C11_4—C10_4—C6_4	109.6 (3)
H3A_2—C3_2—H3B_2	107.6	C9_4—C10_4—C6_4	100.6 (3)
C3_2—C4_2—C5_2	110.2 (4)	C10_4—C11_4—H11A_4	109.5
C3_2—C4_2—C6_2	110.4 (4)	C10_4—C11_4—H11B_4	109.5
C5_2—C4_2—C6_2	112.0 (4)	H11A_4—C11_4—H11B_4	109.5
C3_2—C4_2—H4_2	108.0	C10_4—C11_4—H11C_4	109.5
C5_2—C4_2—H4_2	108.0	H11A_4—C11_4—H11C_4	109.5
C6_2—C4_2—H4_2	108.0	H11B_4—C11_4—H11C_4	109.5
C4_2—C5_2—H5A_2	109.5	O2_4—C12_4—C13_4	109.4 (3)
C4_2—C5_2—H5B_2	109.5	O2_4—C12_4—C10_4	110.6 (3)
H5A_2—C5_2—H5B_2	109.5	C13_4—C12_4—C10_4	111.8 (3)
C4_2—C5_2—H5C_2	109.5	O2_4—C12_4—H12_4	108.3
H5A_2—C5_2—H5C_2	109.5	C13_4—C12_4—H12_4	108.3
H5B_2—C5_2—H5C_2	109.5	C10_4—C12_4—H12_4	108.3
C4_2—C6_2—C10_2	118.9 (3)	C12_4—C13_4—C14_4	115.6 (3)
C4_2—C6_2—C7_2	112.7 (4)	C12_4—C13_4—H13A_4	108.4
C10_2—C6_2—C7_2	102.5 (3)	C14_4—C13_4—H13A_4	108.4
C4_2—C6_2—H6_2	107.4	C12_4—C13_4—H13B_4	108.4
C10_2—C6_2—H6_2	107.4	C14_4—C13_4—H13B_4	108.4
C7_2—C6_2—H6_2	107.4	H13A_4—C13_4—H13B_4	107.5
C8_2—C7_2—C6_2	107.6 (4)	C15_4—C14_4—C13_4	109.5 (3)
C8_2—C7_2—H7A_2	110.2	C15_4—C14_4—C19_4	112.8 (3)
C6_2—C7_2—H7A_2	110.2	C13_4—C14_4—C19_4	113.7 (3)
C8_2—C7_2—H7B_2	110.2	C15_4—C14_4—H14_4	106.8
C6_2—C7_2—H7B_2	110.2	C13_4—C14_4—H14_4	106.8
H7A_2—C7_2—H7B_2	108.5	C19_4—C14_4—H14_4	106.8
C9_2—C8_2—C7_2	103.7 (4)	C9_4—C15_4—C16_4	112.5 (3)
C9_2—C8_2—H8A_2	111.0	C9_4—C15_4—C14_4	108.4 (3)
C7_2—C8_2—H8A_2	111.0	C16_4—C15_4—C14_4	111.4 (3)
C9_2—C8_2—H8B_2	111.0	C9_4—C15_4—H15_4	108.1
C7_2—C8_2—H8B_2	111.0	C16_4—C15_4—H15_4	108.1
H8A_2—C8_2—H8B_2	109.0	C14_4—C15_4—H15_4	108.1
C15_2—C9_2—C8_2	118.3 (4)	C17_4—C16_4—C15_4	111.9 (4)
C15_2—C9_2—C10_2	114.7 (3)	C17_4—C16_4—H16A_4	109.2
C8_2—C9_2—C10_2	104.7 (4)	C15_4—C16_4—H16A_4	109.2
C15_2—C9_2—H9_2	106.1	C17_4—C16_4—H16B_4	109.2
C8_2—C9_2—H9_2	106.1	C15_4—C16_4—H16B_4	109.2
C10_2—C9_2—H9_2	106.1	H16A_4—C16_4—H16B_4	107.9
C12_2—C10_2—C9_2	108.7 (3)	C16_4—C17_4—C18_4	112.2 (4)
C12_2—C10_2—C11_2	108.0 (3)	C16_4—C17_4—H17A_4	109.2
C9_2—C10_2—C11_2	112.3 (3)	C18_4—C17_4—H17A_4	109.2
C12_2—C10_2—C6_2	118.0 (3)	C16_4—C17_4—H17B_4	109.2
C9_2—C10_2—C6_2	100.3 (3)	C18_4—C17_4—H17B_4	109.2
C11_2—C10_2—C6_2	109.4 (3)	H17A_4—C17_4—H17B_4	107.9
C10_2—C11_2—H11A_2	109.5	C17_4—C18_4—C24_4	110.9 (4)
C10_2—C11_2—H11B_2	109.5	C17_4—C18_4—C19_4	111.8 (4)
H11A_2—C11_2—H11B_2	109.5	C24_4—C18_4—C19_4	113.5 (4)
C10_2—C11_2—H11C_2	109.5	C17_4—C18_4—H18_4	106.7
H11A_2—C11_2—H11C_2	109.5	C24_4—C18_4—H18_4	106.7
H11B_2—C11_2—H11C_2	109.5	C19_4—C18_4—H18_4	106.7
O2_2—C12_2—C10_2	110.8 (3)	C21_4—C19_4—C20_4	107.4 (4)
O2_2—C12_2—C13_2	109.7 (3)	C21_4—C19_4—C18_4	107.9 (4)
C10_2—C12_2—C13_2	111.2 (3)	C20_4—C19_4—C18_4	108.9 (4)
O2_2—C12_2—H12_2	108.3	C21_4—C19_4—C14_4	112.0 (3)
C10_2—C12_2—H12_2	108.3	C20_4—C19_4—C14_4	111.1 (4)
C13_2—C12_2—H12_2	108.3	C18_4—C19_4—C14_4	109.6 (3)
C14_2—C13_2—C12_2	115.3 (3)	C19_4—C20_4—H20A_4	109.5
C14_2—C13_2—H13A_2	108.4	C19_4—C20_4—H20B_4	109.5
C12_2—C13_2—H13A_2	108.4	H20A_4—C20_4—H20B_4	109.5
C14_2—C13_2—H13B_2	108.4	C19_4—C20_4—H20C_4	109.5
C12_2—C13_2—H13B_2	108.4	H20A_4—C20_4—H20C_4	109.5
H13A_2—C13_2—H13B_2	107.5	H20B_4—C20_4—H20C_4	109.5
C13_2—C14_2—C15_2	108.6 (3)	C22_4—C21_4—C19_4	113.9 (4)
C13_2—C14_2—C19_2	114.1 (3)	C22_4—C21_4—H21A_4	108.8
C15_2—C14_2—C19_2	112.9 (3)	C19_4—C21_4—H21A_4	108.8
C13_2—C14_2—H14_2	106.9	C22_4—C21_4—H21B_4	108.8
C15_2—C14_2—H14_2	106.9	C19_4—C21_4—H21B_4	108.8
C19_2—C14_2—H14_2	106.9	H21A_4—C21_4—H21B_4	107.7
C9_2—C15_2—C16_2	112.0 (4)	C23_4—C22_4—C21_4	111.2 (4)
C9_2—C15_2—C14_2	107.4 (3)	C23_4—C22_4—H22A_4	109.4
C16_2—C15_2—C14_2	112.6 (4)	C21_4—C22_4—H22A_4	109.4
C9_2—C15_2—H15_2	108.2	C23_4—C22_4—H22B_4	109.4
C16_2—C15_2—H15_2	108.2	C21_4—C22_4—H22B_4	109.4
C14_2—C15_2—H15_2	108.2	H22A_4—C22_4—H22B_4	108.0
C17_2—C16_2—C15_2	111.7 (4)	N1_4—C23_4—C24_4	110.5 (4)
C17_2—C16_2—H16A_2	109.3	N1_4—C23_4—C22_4	111.0 (4)
C15_2—C16_2—H16A_2	109.3	C24_4—C23_4—C22_4	109.9 (4)
C17_2—C16_2—H16B_2	109.3	N1_4—C23_4—H23_4	108.5
C15_2—C16_2—H16B_2	109.3	C24_4—C23_4—H23_4	108.5
H16A_2—C16_2—H16B_2	107.9	C22_4—C23_4—H23_4	108.5
C18_2—C17_2—C16_2	111.5 (4)	C23_4—C24_4—C18_4	114.5 (4)
C18_2—C17_2—H17A_2	109.3	C23_4—C24_4—H24A_4	108.6
C16_2—C17_2—H17A_2	109.3	C18_4—C24_4—H24A_4	108.6
C18_2—C17_2—H17B_2	109.3	C23_4—C24_4—H24B_4	108.6
C16_2—C17_2—H17B_2	109.3	C18_4—C24_4—H24B_4	108.6
H17A_2—C17_2—H17B_2	108.0	H24A_4—C24_4—H24B_4	107.6
C17_2—C18_2—C24_2	110.4 (4)	C25'_4—N1_4—C23_4	121.2 (5)
C17_2—C18_2—C19_2	112.5 (4)	C25_4—N1_4—C23_4	120.7 (5)
C24_2—C18_2—C19_2	111.9 (4)	C25'_4—N1_4—H1N_4	116 (4)
C17_2—C18_2—H18_2	107.3	C25_4—N1_4—H1N_4	116 (4)
C24_2—C18_2—H18_2	107.3	C23_4—N1_4—H1N_4	123 (4)
C19_2—C18_2—H18_2	107.3	O10_4—C25_4—N1_4	122.7 (7)
C21_2—C19_2—C20_2	107.0 (4)	O10_4—C25_4—C26_4	119.5 (8)
C21_2—C19_2—C14_2	112.1 (4)	N1_4—C25_4—C26_4	117.7 (6)
C20_2—C19_2—C14_2	110.9 (4)	C27_4—C26_4—C29_4	108.1 (6)
C21_2—C19_2—C18_2	108.1 (4)	C27_4—C26_4—C25_4	110.4 (6)
C20_2—C19_2—C18_2	108.8 (4)	C29_4—C26_4—C25_4	107.2 (7)
C14_2—C19_2—C18_2	109.8 (4)	C27_4—C26_4—C28_4	110.0 (6)
C19_2—C20_2—H20A_2	109.5	C29_4—C26_4—C28_4	107.6 (6)
C19_2—C20_2—H20B_2	109.5	C25_4—C26_4—C28_4	113.4 (6)
H20A_2—C20_2—H20B_2	109.5	C32_4—C27_4—C26_4	111.4 (6)
C19_2—C20_2—H20C_2	109.5	C32_4—C27_4—H27A_4	109.3
H20A_2—C20_2—H20C_2	109.5	C26_4—C27_4—H27A_4	109.3
H20B_2—C20_2—H20C_2	109.5	C32_4—C27_4—H27B_4	109.3
C22_2—C21_2—C19_2	115.1 (4)	C26_4—C27_4—H27B_4	109.3
C22_2—C21_2—H21A_2	108.5	H27A_4—C27_4—H27B_4	108.0
C19_2—C21_2—H21A_2	108.5	C26_4—C28_4—C34_4	110.0 (6)
C22_2—C21_2—H21B_2	108.5	C26_4—C28_4—H28A_4	109.7
C19_2—C21_2—H21B_2	108.5	C34_4—C28_4—H28A_4	109.7
H21A_2—C21_2—H21B_2	107.5	C26_4—C28_4—H28B_4	109.7
C23_2—C22_2—C21_2	111.9 (4)	C34_4—C28_4—H28B_4	109.7
C23_2—C22_2—H22A_2	109.2	H28A_4—C28_4—H28B_4	108.2
C21_2—C22_2—H22A_2	109.2	C30_4—C29_4—C26_4	111.1 (6)
C23_2—C22_2—H22B_2	109.2	C30_4—C29_4—H29A_4	109.4
C21_2—C22_2—H22B_2	109.2	C26_4—C29_4—H29A_4	109.4
H22A_2—C22_2—H22B_2	107.9	C30_4—C29_4—H29B_4	109.4
N1_2—C23_2—C24_2	110.5 (4)	C26_4—C29_4—H29B_4	109.4
N1_2—C23_2—C22_2	111.5 (4)	H29A_4—C29_4—H29B_4	108.0
C24_2—C23_2—C22_2	110.9 (4)	C35_4—C30_4—C29_4	109.6 (7)
N1_2—C23_2—H23_2	107.9	C35_4—C30_4—C31_4	107.8 (7)
C24_2—C23_2—H23_2	107.9	C29_4—C30_4—C31_4	109.4 (7)
C22_2—C23_2—H23_2	107.9	C35_4—C30_4—H30_4	110.0
C23_2—C24_2—C18_2	114.8 (4)	C29_4—C30_4—H30_4	110.0
C23_2—C24_2—H24A_2	108.6	C31_4—C30_4—H30_4	110.0
C18_2—C24_2—H24A_2	108.6	C32_4—C31_4—C30_4	110.4 (7)
C23_2—C24_2—H24B_2	108.6	C32_4—C31_4—H31A_4	109.6
C18_2—C24_2—H24B_2	108.6	C30_4—C31_4—H31A_4	109.6
H24A_2—C24_2—H24B_2	107.5	C32_4—C31_4—H31B_4	109.6
O3_2—C25_2—N1_2	119.3 (5)	C30_4—C31_4—H31B_4	109.6
O3_2—C25_2—C26_2	121.4 (5)	H31A_4—C31_4—H31B_4	108.1
N1_2—C25_2—C26_2	119.3 (4)	C27_4—C32_4—C31_4	108.7 (6)
C25_2—C26_2—C28_2	115.8 (4)	C27_4—C32_4—C33_4	108.7 (7)
C25_2—C26_2—C27_2	108.4 (4)	C31_4—C32_4—C33_4	108.7 (7)
C28_2—C26_2—C27_2	109.6 (4)	C27_4—C32_4—H32_4	110.2
C25_2—C26_2—C29_2	106.5 (4)	C31_4—C32_4—H32_4	110.2
C28_2—C26_2—C29_2	108.2 (4)	C33_4—C32_4—H32_4	110.2
C27_2—C26_2—C29_2	108.2 (4)	C34_4—C33_4—C32_4	110.9 (7)
C26_2—C27_2—C32_2	110.2 (4)	C34_4—C33_4—H33A_4	109.5
C26_2—C27_2—H27A_2	109.6	C32_4—C33_4—H33A_4	109.5
C32_2—C27_2—H27A_2	109.6	C34_4—C33_4—H33B_4	109.5
C26_2—C27_2—H27B_2	109.6	C32_4—C33_4—H33B_4	109.5
C32_2—C27_2—H27B_2	109.6	H33A_4—C33_4—H33B_4	108.0
H27A_2—C27_2—H27B_2	108.1	C35_4—C34_4—C33_4	109.4 (7)
C26_2—C28_2—C34_2	110.0 (4)	C35_4—C34_4—C28_4	109.6 (7)
C26_2—C28_2—H28A_2	109.7	C33_4—C34_4—C28_4	108.1 (7)
C34_2—C28_2—H28A_2	109.7	C35_4—C34_4—H34_4	109.9
C26_2—C28_2—H28B_2	109.7	C33_4—C34_4—H34_4	109.9
C34_2—C28_2—H28B_2	109.7	C28_4—C34_4—H34_4	109.9
H28A_2—C28_2—H28B_2	108.2	C30_4—C35_4—C34_4	110.8 (7)
C30_2—C29_2—C26_2	110.2 (4)	C30_4—C35_4—H35A_4	109.5
C30_2—C29_2—H29A_2	109.6	C34_4—C35_4—H35A_4	109.5
C26_2—C29_2—H29A_2	109.6	C30_4—C35_4—H35B_4	109.5
C30_2—C29_2—H29B_2	109.6	C34_4—C35_4—H35B_4	109.5
C26_2—C29_2—H29B_2	109.6	H35A_4—C35_4—H35B_4	108.1
H29A_2—C29_2—H29B_2	108.1	O10'_4—C25'_4—N1_4	122.6 (8)
C31_2—C30_2—C35_2	109.8 (4)	O10'_4—C25'_4—C26'_4	119.9 (9)
C31_2—C30_2—C29_2	108.8 (4)	N1_4—C25'_4—C26'_4	117.2 (6)
C35_2—C30_2—C29_2	109.7 (4)	C29'_4—C26'_4—C27'_4	109.4 (6)
C31_2—C30_2—H30_2	109.5	C29'_4—C26'_4—C25'_4	107.5 (6)
C35_2—C30_2—H30_2	109.5	C27'_4—C26'_4—C25'_4	110.5 (7)
C29_2—C30_2—H30_2	109.5	C29'_4—C26'_4—C28'_4	107.3 (6)
C30_2—C31_2—C32_2	109.9 (4)	C27'_4—C26'_4—C28'_4	109.4 (6)
C30_2—C31_2—H31A_2	109.7	C25'_4—C26'_4—C28'_4	112.7 (7)
C32_2—C31_2—H31A_2	109.7	C32'_4—C27'_4—C26'_4	110.9 (6)
C30_2—C31_2—H31B_2	109.7	C32'_4—C27'_4—H27C_4	109.5
C32_2—C31_2—H31B_2	109.7	C26'_4—C27'_4—H27C_4	109.5
H31A_2—C31_2—H31B_2	108.2	C32'_4—C27'_4—H27D_4	109.5
C31_2—C32_2—C33_2	110.0 (4)	C26'_4—C27'_4—H27D_4	109.5
C31_2—C32_2—C27_2	108.8 (5)	H27C_4—C27'_4—H27D_4	108.0
C33_2—C32_2—C27_2	108.5 (4)	C34'_4—C28'_4—C26'_4	110.3 (6)
C31_2—C32_2—H32_2	109.8	C34'_4—C28'_4—H28C_4	109.6
C33_2—C32_2—H32_2	109.8	C26'_4—C28'_4—H28C_4	109.6
C27_2—C32_2—H32_2	109.8	C34'_4—C28'_4—H28D_4	109.6
C34_2—C33_2—C32_2	109.9 (4)	C26'_4—C28'_4—H28D_4	109.6
C34_2—C33_2—H33A_2	109.7	H28C_4—C28'_4—H28D_4	108.1
C32_2—C33_2—H33A_2	109.7	C26'_4—C29'_4—C30'_4	110.8 (6)
C34_2—C33_2—H33B_2	109.7	C26'_4—C29'_4—H29C_4	109.5
C32_2—C33_2—H33B_2	109.7	C30'_4—C29'_4—H29C_4	109.5
H33A_2—C33_2—H33B_2	108.2	C26'_4—C29'_4—H29D_4	109.5
C33_2—C34_2—C35_2	109.5 (4)	C30'_4—C29'_4—H29D_4	109.5
C33_2—C34_2—C28_2	110.1 (4)	H29C_4—C29'_4—H29D_4	108.1
C35_2—C34_2—C28_2	109.5 (4)	C35'_4—C30'_4—C29'_4	109.5 (7)
C33_2—C34_2—H34_2	109.2	C35'_4—C30'_4—C31'_4	109.0 (7)
C35_2—C34_2—H34_2	109.2	C29'_4—C30'_4—C31'_4	109.2 (6)
C28_2—C34_2—H34_2	109.2	C35'_4—C30'_4—H30'_4	109.7
C30_2—C35_2—C34_2	109.5 (4)	C29'_4—C30'_4—H30'_4	109.7
C30_2—C35_2—H35A_2	109.8	C31'_4—C30'_4—H30'_4	109.7
C34_2—C35_2—H35A_2	109.8	C30'_4—C31'_4—C32'_4	109.6 (7)
C30_2—C35_2—H35B_2	109.8	C30'_4—C31'_4—H31C_4	109.8
C34_2—C35_2—H35B_2	109.8	C32'_4—C31'_4—H31C_4	109.8
H35A_2—C35_2—H35B_2	108.2	C30'_4—C31'_4—H31D_4	109.8
C1_3—O1_3—H1_3	99 (4)	C32'_4—C31'_4—H31D_4	109.8
C12_3—O2_3—H2_3	106 (3)	H31C_4—C31'_4—H31D_4	108.2
C25_3—N1_3—C23_3	121.8 (4)	C27'_4—C32'_4—C33'_4	109.4 (7)
C25_3—N1_3—H1N_3	120 (4)	C27'_4—C32'_4—C31'_4	107.7 (7)
C23_3—N1_3—H1N_3	115 (4)	C33'_4—C32'_4—C31'_4	109.2 (7)
O1_3—C1_3—C2_3	113.9 (4)	C27'_4—C32'_4—H32'_4	110.2
O1_3—C1_3—H1A_3	108.8	C33'_4—C32'_4—H32'_4	110.2
C2_3—C1_3—H1A_3	108.8	C31'_4—C32'_4—H32'_4	110.2
O1_3—C1_3—H1B_3	108.8	C34'_4—C33'_4—C32'_4	111.2 (7)
C2_3—C1_3—H1B_3	108.8	C34'_4—C33'_4—H33C_4	109.4
H1A_3—C1_3—H1B_3	107.7	C32'_4—C33'_4—H33C_4	109.4
C1_3—C2_3—C3_3	110.9 (4)	C34'_4—C33'_4—H33D_4	109.4
C1_3—C2_3—H2A_3	109.5	C32'_4—C33'_4—H33D_4	109.4
C3_3—C2_3—H2A_3	109.5	H33C_4—C33'_4—H33D_4	108.0
C1_3—C2_3—H2B_3	109.5	C33'_4—C34'_4—C35'_4	108.7 (7)
C3_3—C2_3—H2B_3	109.5	C33'_4—C34'_4—C28'_4	108.1 (7)
H2A_3—C2_3—H2B_3	108.1	C35'_4—C34'_4—C28'_4	110.1 (7)
C4_3—C3_3—C2_3	115.4 (4)	C33'_4—C34'_4—H34'_4	110.0
C4_3—C3_3—H3A_3	108.4	C35'_4—C34'_4—H34'_4	110.0
C2_3—C3_3—H3A_3	108.4	C28'_4—C34'_4—H34'_4	110.0
C4_3—C3_3—H3B_3	108.4	C30'_4—C35'_4—C34'_4	110.6 (7)
C2_3—C3_3—H3B_3	108.4	C30'_4—C35'_4—H35C_4	109.5
H3A_3—C3_3—H3B_3	107.5	C34'_4—C35'_4—H35C_4	109.5
C3_3—C4_3—C6_3	111.4 (3)	C30'_4—C35'_4—H35D_4	109.5
C3_3—C4_3—C5_3	109.5 (4)	C34'_4—C35'_4—H35D_4	109.5
C6_3—C4_3—C5_3	111.5 (3)	H35C_4—C35'_4—H35D_4	108.1
C3_3—C4_3—H4_3	108.1	H1W—O1W—H2W	110 (8)
C6_3—C4_3—H4_3	108.1		
			
O3_1—C25_1—N1_1—C23_1	−4.8 (13)	C7_3—C8_3—C9_3—C15_3	−158.6 (4)
C26_1—C25_1—N1_1—C23_1	170.1 (6)	C7_3—C8_3—C9_3—C10_3	−30.4 (4)
C24_1—C23_1—N1_1—C25_1	−140.9 (8)	C15_3—C9_3—C10_3—C11_3	58.8 (5)
C22_1—C23_1—N1_1—C25_1	94.1 (9)	C8_3—C9_3—C10_3—C11_3	−72.5 (4)
O3_1—C25_1—C26_1—C27_1	−4.5 (9)	C15_3—C9_3—C10_3—C12_3	−62.0 (4)
N1_1—C25_1—C26_1—C27_1	−179.2 (7)	C8_3—C9_3—C10_3—C12_3	166.7 (3)
O3_1—C25_1—C26_1—C28_1	115.3 (7)	C15_3—C9_3—C10_3—C6_3	175.8 (3)
N1_1—C25_1—C26_1—C28_1	−59.4 (8)	C8_3—C9_3—C10_3—C6_3	44.5 (4)
O3_1—C25_1—C26_1—C29_1	−122.3 (7)	C4_3—C6_3—C10_3—C11_3	−47.8 (5)
N1_1—C25_1—C26_1—C29_1	63.0 (8)	C7_3—C6_3—C10_3—C11_3	78.7 (4)
C27_1—C26_1—C25'_1—O3'_1	−48 (3)	C4_3—C6_3—C10_3—C12_3	77.4 (5)
C28_1—C26_1—C25'_1—O3'_1	67 (2)	C7_3—C6_3—C10_3—C12_3	−156.1 (3)
C29_1—C26_1—C25'_1—O3'_1	−176.5 (17)	C4_3—C6_3—C10_3—C9_3	−167.3 (3)
C27_1—C26_1—C25'_1—N1'_1	136 (2)	C7_3—C6_3—C10_3—C9_3	−40.8 (4)
C28_1—C26_1—C25'_1—N1'_1	−110 (3)	C11_3—C10_3—C12_3—O2_3	172.8 (3)
C29_1—C26_1—C25'_1—N1'_1	7 (3)	C9_3—C10_3—C12_3—O2_3	−64.0 (4)
O3'_1—C25'_1—N1'_1—C23_1	−8 (4)	C6_3—C10_3—C12_3—O2_3	47.5 (5)
C26_1—C25'_1—N1'_1—C23_1	167.8 (16)	C11_3—C10_3—C12_3—C13_3	−66.6 (4)
C24_1—C23_1—N1'_1—C25'_1	−93 (3)	C9_3—C10_3—C12_3—C13_3	56.6 (4)
C22_1—C23_1—N1'_1—C25'_1	143 (2)	C6_3—C10_3—C12_3—C13_3	168.1 (3)
O1_1—C1_1—C2_1—C3_1	154.6 (4)	O2_3—C12_3—C13_3—C14_3	66.9 (4)
C1_1—C2_1—C3_1—C4_1	63.1 (5)	C10_3—C12_3—C13_3—C14_3	−54.5 (5)
C2_1—C3_1—C4_1—C6_1	−156.5 (4)	C12_3—C13_3—C14_3—C15_3	51.9 (5)
C2_1—C3_1—C4_1—C5_1	77.6 (5)	C12_3—C13_3—C14_3—C19_3	−179.3 (4)
C5_1—C4_1—C6_1—C10_1	−59.8 (5)	C8_3—C9_3—C15_3—C16_3	−55.7 (5)
C3_1—C4_1—C6_1—C10_1	176.3 (4)	C10_3—C9_3—C15_3—C16_3	−179.3 (3)
C5_1—C4_1—C6_1—C7_1	−179.6 (4)	C8_3—C9_3—C15_3—C14_3	−177.1 (4)
C3_1—C4_1—C6_1—C7_1	56.5 (5)	C10_3—C9_3—C15_3—C14_3	59.3 (4)
C4_1—C6_1—C7_1—C8_1	147.6 (4)	C13_3—C14_3—C15_3—C9_3	−52.0 (4)
C10_1—C6_1—C7_1—C8_1	18.6 (5)	C19_3—C14_3—C15_3—C9_3	178.8 (3)
C6_1—C7_1—C8_1—C9_1	9.4 (5)	C13_3—C14_3—C15_3—C16_3	−174.4 (4)
C7_1—C8_1—C9_1—C15_1	−163.2 (4)	C19_3—C14_3—C15_3—C16_3	56.4 (4)
C7_1—C8_1—C9_1—C10_1	−34.7 (5)	C9_3—C15_3—C16_3—C17_3	−174.2 (4)
C15_1—C9_1—C10_1—C12_1	−57.3 (5)	C14_3—C15_3—C16_3—C17_3	−53.9 (5)
C8_1—C9_1—C10_1—C12_1	171.8 (4)	C15_3—C16_3—C17_3—C18_3	53.4 (5)
C15_1—C9_1—C10_1—C11_1	61.8 (5)	C16_3—C17_3—C18_3—C24_3	72.6 (5)
C8_1—C9_1—C10_1—C11_1	−69.1 (5)	C16_3—C17_3—C18_3—C19_3	−53.6 (5)
C15_1—C9_1—C10_1—C6_1	177.4 (4)	C24_3—C18_3—C19_3—C20_3	167.2 (4)
C8_1—C9_1—C10_1—C6_1	46.6 (4)	C17_3—C18_3—C19_3—C20_3	−67.9 (5)
C4_1—C6_1—C10_1—C9_1	−163.7 (4)	C24_3—C18_3—C19_3—C21_3	50.8 (5)
C7_1—C6_1—C10_1—C9_1	−39.0 (4)	C17_3—C18_3—C19_3—C21_3	175.7 (4)
C4_1—C6_1—C10_1—C12_1	77.9 (5)	C24_3—C18_3—C19_3—C14_3	−70.8 (5)
C7_1—C6_1—C10_1—C12_1	−157.5 (4)	C17_3—C18_3—C19_3—C14_3	54.1 (5)
C4_1—C6_1—C10_1—C11_1	−45.4 (5)	C13_3—C14_3—C19_3—C20_3	−63.8 (5)
C7_1—C6_1—C10_1—C11_1	79.2 (4)	C15_3—C14_3—C19_3—C20_3	64.7 (5)
C9_1—C10_1—C12_1—O2_1	−70.7 (4)	C13_3—C14_3—C19_3—C21_3	55.8 (5)
C11_1—C10_1—C12_1—O2_1	167.3 (3)	C15_3—C14_3—C19_3—C21_3	−175.7 (3)
C6_1—C10_1—C12_1—O2_1	43.4 (5)	C13_3—C14_3—C19_3—C18_3	175.1 (4)
C9_1—C10_1—C12_1—C13_1	51.6 (4)	C15_3—C14_3—C19_3—C18_3	−56.4 (5)
C11_1—C10_1—C12_1—C13_1	−70.4 (4)	C20_3—C19_3—C21_3—C22_3	−175.2 (4)
C6_1—C10_1—C12_1—C13_1	165.7 (4)	C18_3—C19_3—C21_3—C22_3	−57.2 (5)
O2_1—C12_1—C13_1—C14_1	68.6 (4)	C14_3—C19_3—C21_3—C22_3	62.8 (5)
C10_1—C12_1—C13_1—C14_1	−53.6 (5)	C19_3—C21_3—C22_3—C23_3	58.6 (5)
C12_1—C13_1—C14_1—C15_1	55.3 (5)	C25_3—N1_3—C23_3—C22_3	156.3 (4)
C12_1—C13_1—C14_1—C19_1	−177.1 (3)	C25_3—N1_3—C23_3—C24_3	−80.8 (5)
C8_1—C9_1—C15_1—C16_1	−52.0 (6)	C21_3—C22_3—C23_3—N1_3	73.5 (5)
C10_1—C9_1—C15_1—C16_1	−175.6 (4)	C21_3—C22_3—C23_3—C24_3	−50.6 (5)
C8_1—C9_1—C15_1—C14_1	−176.3 (4)	C17_3—C18_3—C24_3—C23_3	−175.0 (4)
C10_1—C9_1—C15_1—C14_1	60.0 (5)	C19_3—C18_3—C24_3—C23_3	−49.2 (5)
C13_1—C14_1—C15_1—C16_1	−179.3 (4)	N1_3—C23_3—C24_3—C18_3	−74.0 (5)
C19_1—C14_1—C15_1—C16_1	51.9 (5)	C22_3—C23_3—C24_3—C18_3	47.7 (5)
C13_1—C14_1—C15_1—C9_1	−56.1 (5)	C23_3—N1_3—C25_3—O3_3	8.9 (7)
C19_1—C14_1—C15_1—C9_1	175.1 (4)	C23_3—N1_3—C25_3—C26_3	−170.1 (4)
C9_1—C15_1—C16_1—C17_1	−172.1 (4)	O3_3—C25_3—C26_3—C29_3	−62.3 (5)
C14_1—C15_1—C16_1—C17_1	−50.3 (6)	N1_3—C25_3—C26_3—C29_3	116.7 (4)
C15_1—C16_1—C17_1—C18_1	53.2 (6)	O3_3—C25_3—C26_3—C28_3	177.0 (4)
C16_1—C17_1—C18_1—C24_1	67.8 (6)	N1_3—C25_3—C26_3—C28_3	−4.0 (6)
C16_1—C17_1—C18_1—C19_1	−58.1 (6)	O3_3—C25_3—C26_3—C27_3	54.8 (5)
C24_1—C18_1—C19_1—C21_1	55.8 (5)	N1_3—C25_3—C26_3—C27_3	−126.2 (4)
C17_1—C18_1—C19_1—C21_1	−179.0 (4)	C25_3—C26_3—C27_3—C32_3	−175.2 (4)
C24_1—C18_1—C19_1—C20_1	171.1 (4)	C29_3—C26_3—C27_3—C32_3	−58.7 (5)
C17_1—C18_1—C19_1—C20_1	−63.8 (5)	C28_3—C26_3—C27_3—C32_3	58.5 (5)
C24_1—C18_1—C19_1—C14_1	−66.7 (5)	C25_3—C26_3—C28_3—C34_3	178.8 (4)
C17_1—C18_1—C19_1—C14_1	58.5 (5)	C29_3—C26_3—C28_3—C34_3	58.6 (5)
C13_1—C14_1—C19_1—C21_1	59.9 (5)	C27_3—C26_3—C28_3—C34_3	−58.6 (5)
C15_1—C14_1—C19_1—C21_1	−174.1 (4)	C25_3—C26_3—C29_3—C30_3	176.1 (4)
C13_1—C14_1—C19_1—C18_1	179.0 (4)	C28_3—C26_3—C29_3—C30_3	−58.8 (5)
C15_1—C14_1—C19_1—C18_1	−55.0 (5)	C27_3—C26_3—C29_3—C30_3	58.5 (5)
C13_1—C14_1—C19_1—C20_1	−59.3 (5)	C26_3—C29_3—C30_3—C31_3	−59.8 (5)
C15_1—C14_1—C19_1—C20_1	66.7 (5)	C26_3—C29_3—C30_3—C35_3	59.9 (5)
C18_1—C19_1—C21_1—C22_1	−57.5 (5)	C35_3—C30_3—C31_3—C32_3	−59.5 (5)
C20_1—C19_1—C21_1—C22_1	−175.5 (4)	C29_3—C30_3—C31_3—C32_3	60.0 (5)
C14_1—C19_1—C21_1—C22_1	62.3 (5)	C30_3—C31_3—C32_3—C33_3	59.7 (5)
C19_1—C21_1—C22_1—C23_1	54.0 (5)	C30_3—C31_3—C32_3—C27_3	−60.2 (5)
N1_1—C23_1—C22_1—C21_1	75.5 (6)	C26_3—C27_3—C32_3—C31_3	60.0 (5)
N1'_1—C23_1—C22_1—C21_1	83.6 (10)	C26_3—C27_3—C32_3—C33_3	−60.1 (5)
C24_1—C23_1—C22_1—C21_1	−46.5 (5)	C31_3—C32_3—C33_3—C34_3	−59.1 (5)
N1_1—C23_1—C24_1—C18_1	−78.5 (6)	C27_3—C32_3—C33_3—C34_3	60.7 (5)
N1'_1—C23_1—C24_1—C18_1	−70.5 (9)	C32_3—C33_3—C34_3—C35_3	59.9 (5)
C22_1—C23_1—C24_1—C18_1	46.9 (6)	C32_3—C33_3—C34_3—C28_3	−61.3 (5)
C17_1—C18_1—C24_1—C23_1	−178.4 (4)	C26_3—C28_3—C34_3—C35_3	−60.7 (5)
C19_1—C18_1—C24_1—C23_1	−52.8 (5)	C26_3—C28_3—C34_3—C33_3	60.9 (5)
C28_1—C26_1—C27_1—C32_1	59.2 (5)	C33_3—C34_3—C35_3—C30_3	−60.1 (5)
C25'_1—C26_1—C27_1—C32_1	166.7 (10)	C28_3—C34_3—C35_3—C30_3	61.0 (5)
C29_1—C26_1—C27_1—C32_1	−59.6 (5)	C31_3—C30_3—C35_3—C34_3	59.5 (5)
C25_1—C26_1—C27_1—C32_1	−178.3 (4)	C29_3—C30_3—C35_3—C34_3	−60.3 (5)
C27_1—C26_1—C28_1—C34_1	−59.4 (5)	O1_4—C1_4—C2_4—C3_4	175.0 (4)
C25'_1—C26_1—C28_1—C34_1	−176.8 (7)	C1_4—C2_4—C3_4—C4_4	−175.4 (4)
C29_1—C26_1—C28_1—C34_1	59.2 (5)	C2_4—C3_4—C4_4—C5_4	68.6 (5)
C25_1—C26_1—C28_1—C34_1	−177.9 (4)	C2_4—C3_4—C4_4—C6_4	−168.3 (4)
C27_1—C26_1—C29_1—C30_1	60.1 (5)	C5_4—C4_4—C6_4—C10_4	−64.2 (5)
C28_1—C26_1—C29_1—C30_1	−58.7 (5)	C3_4—C4_4—C6_4—C10_4	173.8 (3)
C25'_1—C26_1—C29_1—C30_1	−169.3 (12)	C5_4—C4_4—C6_4—C7_4	174.9 (4)
C25_1—C26_1—C29_1—C30_1	176.5 (4)	C3_4—C4_4—C6_4—C7_4	53.0 (5)
C26_1—C29_1—C30_1—C35_1	59.4 (5)	C4_4—C6_4—C7_4—C8_4	152.5 (4)
C26_1—C29_1—C30_1—C31_1	−60.7 (5)	C10_4—C6_4—C7_4—C8_4	23.3 (4)
C35_1—C30_1—C31_1—C32_1	−59.8 (5)	C6_4—C7_4—C8_4—C9_4	4.5 (4)
C29_1—C30_1—C31_1—C32_1	60.6 (5)	C7_4—C8_4—C9_4—C15_4	−159.5 (4)
C30_1—C31_1—C32_1—C33_1	59.3 (5)	C7_4—C8_4—C9_4—C10_4	−30.9 (4)
C30_1—C31_1—C32_1—C27_1	−59.8 (5)	C15_4—C9_4—C10_4—C12_4	−59.1 (4)
C26_1—C27_1—C32_1—C31_1	59.8 (5)	C8_4—C9_4—C10_4—C12_4	170.5 (3)
C26_1—C27_1—C32_1—C33_1	−59.0 (5)	C15_4—C9_4—C10_4—C11_4	59.3 (5)
C31_1—C32_1—C33_1—C34_1	−60.0 (5)	C8_4—C9_4—C10_4—C11_4	−71.1 (4)
C27_1—C32_1—C33_1—C34_1	58.9 (5)	C15_4—C9_4—C10_4—C6_4	175.7 (3)
C32_1—C33_1—C34_1—C35_1	60.7 (5)	C8_4—C9_4—C10_4—C6_4	45.3 (4)
C32_1—C33_1—C34_1—C28_1	−59.8 (5)	C4_4—C6_4—C10_4—C12_4	75.9 (5)
C26_1—C28_1—C34_1—C33_1	60.4 (5)	C7_4—C6_4—C10_4—C12_4	−158.3 (4)
C26_1—C28_1—C34_1—C35_1	−60.3 (5)	C4_4—C6_4—C10_4—C11_4	−49.0 (5)
C31_1—C30_1—C35_1—C34_1	59.9 (5)	C7_4—C6_4—C10_4—C11_4	76.8 (4)
C29_1—C30_1—C35_1—C34_1	−59.9 (5)	C4_4—C6_4—C10_4—C9_4	−167.3 (4)
C33_1—C34_1—C35_1—C30_1	−60.6 (5)	C7_4—C6_4—C10_4—C9_4	−41.6 (4)
C28_1—C34_1—C35_1—C30_1	60.2 (5)	C11_4—C10_4—C12_4—O2_4	168.5 (3)
O1_2—C1_2—C2_2—C3_2	−177.7 (4)	C9_4—C10_4—C12_4—O2_4	−70.4 (4)
C1_2—C2_2—C3_2—C4_2	170.1 (4)	C6_4—C10_4—C12_4—O2_4	42.7 (5)
C2_2—C3_2—C4_2—C5_2	64.9 (5)	C11_4—C10_4—C12_4—C13_4	−69.4 (4)
C2_2—C3_2—C4_2—C6_2	−170.7 (4)	C9_4—C10_4—C12_4—C13_4	51.8 (4)
C3_2—C4_2—C6_2—C10_2	172.5 (4)	C6_4—C10_4—C12_4—C13_4	164.9 (3)
C5_2—C4_2—C6_2—C10_2	−64.2 (5)	O2_4—C12_4—C13_4—C14_4	70.2 (4)
C3_2—C4_2—C6_2—C7_2	52.5 (5)	C10_4—C12_4—C13_4—C14_4	−52.7 (5)
C5_2—C4_2—C6_2—C7_2	175.8 (4)	C12_4—C13_4—C14_4—C15_4	53.0 (5)
C4_2—C6_2—C7_2—C8_2	150.0 (4)	C12_4—C13_4—C14_4—C19_4	−179.9 (4)
C10_2—C6_2—C7_2—C8_2	21.0 (4)	C8_4—C9_4—C15_4—C16_4	−51.8 (5)
C6_2—C7_2—C8_2—C9_2	6.9 (5)	C10_4—C9_4—C15_4—C16_4	−175.2 (4)
C7_2—C8_2—C9_2—C15_2	−162.2 (4)	C8_4—C9_4—C15_4—C14_4	−175.5 (3)
C7_2—C8_2—C9_2—C10_2	−33.0 (4)	C10_4—C9_4—C15_4—C14_4	61.2 (4)
C15_2—C9_2—C10_2—C12_2	−58.1 (4)	C13_4—C14_4—C15_4—C9_4	−54.2 (4)
C8_2—C9_2—C10_2—C12_2	170.5 (3)	C19_4—C14_4—C15_4—C9_4	178.1 (3)
C15_2—C9_2—C10_2—C11_2	61.3 (5)	C13_4—C14_4—C15_4—C16_4	−178.5 (3)
C8_2—C9_2—C10_2—C11_2	−70.1 (4)	C19_4—C14_4—C15_4—C16_4	53.8 (5)
C15_2—C9_2—C10_2—C6_2	177.4 (3)	C9_4—C15_4—C16_4—C17_4	−174.9 (4)
C8_2—C9_2—C10_2—C6_2	46.0 (4)	C14_4—C15_4—C16_4—C17_4	−52.9 (5)
C4_2—C6_2—C10_2—C12_2	77.1 (5)	C15_4—C16_4—C17_4—C18_4	54.3 (5)
C7_2—C6_2—C10_2—C12_2	−157.8 (4)	C16_4—C17_4—C18_4—C24_4	72.1 (5)
C4_2—C6_2—C10_2—C9_2	−165.2 (4)	C16_4—C17_4—C18_4—C19_4	−55.7 (5)
C7_2—C6_2—C10_2—C9_2	−40.1 (4)	C17_4—C18_4—C19_4—C21_4	176.8 (4)
C4_2—C6_2—C10_2—C11_2	−46.9 (5)	C24_4—C18_4—C19_4—C21_4	50.4 (5)
C7_2—C6_2—C10_2—C11_2	78.2 (4)	C17_4—C18_4—C19_4—C20_4	−67.0 (5)
C9_2—C10_2—C12_2—O2_2	−72.9 (4)	C24_4—C18_4—C19_4—C20_4	166.6 (4)
C11_2—C10_2—C12_2—O2_2	165.1 (3)	C17_4—C18_4—C19_4—C14_4	54.7 (5)
C6_2—C10_2—C12_2—O2_2	40.4 (5)	C24_4—C18_4—C19_4—C14_4	−71.7 (5)
C9_2—C10_2—C12_2—C13_2	49.4 (4)	C15_4—C14_4—C19_4—C21_4	−174.0 (4)
C11_2—C10_2—C12_2—C13_2	−72.6 (4)	C13_4—C14_4—C19_4—C21_4	60.6 (5)
C6_2—C10_2—C12_2—C13_2	162.7 (3)	C15_4—C14_4—C19_4—C20_4	66.0 (4)
O2_2—C12_2—C13_2—C14_2	71.0 (4)	C13_4—C14_4—C19_4—C20_4	−59.4 (5)
C10_2—C12_2—C13_2—C14_2	−52.0 (5)	C15_4—C14_4—C19_4—C18_4	−54.3 (5)
C12_2—C13_2—C14_2—C15_2	55.6 (4)	C13_4—C14_4—C19_4—C18_4	−179.7 (4)
C12_2—C13_2—C14_2—C19_2	−177.5 (4)	C20_4—C19_4—C21_4—C22_4	−172.2 (4)
C8_2—C9_2—C15_2—C16_2	−48.9 (5)	C18_4—C19_4—C21_4—C22_4	−55.0 (5)
C10_2—C9_2—C15_2—C16_2	−173.4 (4)	C14_4—C19_4—C21_4—C22_4	65.6 (5)
C8_2—C9_2—C15_2—C14_2	−173.0 (4)	C19_4—C21_4—C22_4—C23_4	58.9 (5)
C10_2—C9_2—C15_2—C14_2	62.5 (4)	C21_4—C22_4—C23_4—N1_4	68.2 (5)
C13_2—C14_2—C15_2—C9_2	−57.5 (4)	C21_4—C22_4—C23_4—C24_4	−54.3 (5)
C19_2—C14_2—C15_2—C9_2	174.9 (3)	N1_4—C23_4—C24_4—C18_4	−70.9 (5)
C13_2—C14_2—C15_2—C16_2	178.7 (3)	C22_4—C23_4—C24_4—C18_4	51.8 (5)
C19_2—C14_2—C15_2—C16_2	51.2 (5)	C17_4—C18_4—C24_4—C23_4	−178.4 (4)
C9_2—C15_2—C16_2—C17_2	−173.9 (4)	C19_4—C18_4—C24_4—C23_4	−51.6 (5)
C14_2—C15_2—C16_2—C17_2	−52.7 (5)	C24_4—C23_4—N1_4—C25'_4	−156.4 (9)
C15_2—C16_2—C17_2—C18_2	55.6 (5)	C22_4—C23_4—N1_4—C25'_4	81.5 (9)
C16_2—C17_2—C18_2—C24_2	68.6 (5)	C24_4—C23_4—N1_4—C25_4	−167.6 (9)
C16_2—C17_2—C18_2—C19_2	−57.2 (5)	C22_4—C23_4—N1_4—C25_4	70.3 (9)
C13_2—C14_2—C19_2—C21_2	64.3 (5)	C23_4—N1_4—C25_4—O10_4	6 (2)
C15_2—C14_2—C19_2—C21_2	−171.1 (3)	C23_4—N1_4—C25_4—C26_4	−177.4 (7)
C13_2—C14_2—C19_2—C20_2	−55.2 (5)	O10_4—C25_4—C26_4—C27_4	43.6 (17)
C15_2—C14_2—C19_2—C20_2	69.4 (5)	N1_4—C25_4—C26_4—C27_4	−133.7 (11)
C13_2—C14_2—C19_2—C18_2	−175.5 (3)	O10_4—C25_4—C26_4—C29_4	−74.0 (16)
C15_2—C14_2—C19_2—C18_2	−50.9 (5)	N1_4—C25_4—C26_4—C29_4	108.8 (12)
C17_2—C18_2—C19_2—C21_2	176.7 (4)	O10_4—C25_4—C26_4—C28_4	167.4 (14)
C24_2—C18_2—C19_2—C21_2	51.7 (5)	N1_4—C25_4—C26_4—C28_4	−9.8 (14)
C17_2—C18_2—C19_2—C20_2	−67.4 (5)	C29_4—C26_4—C27_4—C32_4	−59.9 (8)
C24_2—C18_2—C19_2—C20_2	167.6 (4)	C25_4—C26_4—C27_4—C32_4	−176.8 (8)
C17_2—C18_2—C19_2—C14_2	54.2 (5)	C28_4—C26_4—C27_4—C32_4	57.4 (8)
C24_2—C18_2—C19_2—C14_2	−70.8 (5)	C27_4—C26_4—C28_4—C34_4	−57.8 (8)
C20_2—C19_2—C21_2—C22_2	−171.1 (4)	C29_4—C26_4—C28_4—C34_4	59.7 (8)
C14_2—C19_2—C21_2—C22_2	67.1 (5)	C25_4—C26_4—C28_4—C34_4	178.1 (8)
C18_2—C19_2—C21_2—C22_2	−54.1 (5)	C27_4—C26_4—C29_4—C30_4	58.7 (8)
C19_2—C21_2—C22_2—C23_2	55.1 (5)	C25_4—C26_4—C29_4—C30_4	177.6 (6)
C25_2—N1_2—C23_2—C24_2	−158.6 (4)	C28_4—C26_4—C29_4—C30_4	−60.1 (8)
C25_2—N1_2—C23_2—C22_2	77.6 (6)	C26_4—C29_4—C30_4—C35_4	59.6 (9)
C21_2—C22_2—C23_2—N1_2	72.6 (5)	C26_4—C29_4—C30_4—C31_4	−58.4 (9)
C21_2—C22_2—C23_2—C24_2	−51.0 (5)	C35_4—C30_4—C31_4—C32_4	−61.0 (8)
N1_2—C23_2—C24_2—C18_2	−72.1 (5)	C29_4—C30_4—C31_4—C32_4	58.1 (8)
C22_2—C23_2—C24_2—C18_2	52.1 (5)	C26_4—C27_4—C32_4—C31_4	60.3 (8)
C17_2—C18_2—C24_2—C23_2	−179.6 (4)	C26_4—C27_4—C32_4—C33_4	−57.8 (8)
C19_2—C18_2—C24_2—C23_2	−53.5 (5)	C30_4—C31_4—C32_4—C27_4	−58.7 (8)
C23_2—N1_2—C25_2—O3_2	−1.8 (8)	C30_4—C31_4—C32_4—C33_4	59.4 (8)
C23_2—N1_2—C25_2—C26_2	−179.3 (4)	C27_4—C32_4—C33_4—C34_4	60.4 (9)
O3_2—C25_2—C26_2—C28_2	156.4 (5)	C31_4—C32_4—C33_4—C34_4	−57.8 (9)
N1_2—C25_2—C26_2—C28_2	−26.2 (7)	C32_4—C33_4—C34_4—C35_4	57.9 (9)
O3_2—C25_2—C26_2—C27_2	32.8 (7)	C32_4—C33_4—C34_4—C28_4	−61.3 (9)
N1_2—C25_2—C26_2—C27_2	−149.7 (5)	C26_4—C28_4—C34_4—C35_4	−59.6 (8)
O3_2—C25_2—C26_2—C29_2	−83.3 (6)	C26_4—C28_4—C34_4—C33_4	59.5 (9)
N1_2—C25_2—C26_2—C29_2	94.1 (5)	C29_4—C30_4—C35_4—C34_4	−58.0 (9)
C25_2—C26_2—C27_2—C32_2	−174.2 (4)	C31_4—C30_4—C35_4—C34_4	61.0 (9)
C28_2—C26_2—C27_2—C32_2	58.6 (5)	C33_4—C34_4—C35_4—C30_4	−60.0 (9)
C29_2—C26_2—C27_2—C32_2	−59.1 (5)	C28_4—C34_4—C35_4—C30_4	58.3 (9)
C25_2—C26_2—C28_2—C34_2	179.2 (4)	C23_4—N1_4—C25'_4—O10'_4	14 (2)
C27_2—C26_2—C28_2—C34_2	−57.9 (5)	C23_4—N1_4—C25'_4—C26'_4	−172.0 (7)
C29_2—C26_2—C28_2—C34_2	59.9 (5)	O10'_4—C25'_4—C26'_4—C29'_4	−25.6 (18)
C25_2—C26_2—C29_2—C30_2	175.9 (4)	N1_4—C25'_4—C26'_4—C29'_4	160.3 (11)
C28_2—C26_2—C29_2—C30_2	−59.0 (5)	O10'_4—C25'_4—C26'_4—C27'_4	93.6 (17)
C27_2—C26_2—C29_2—C30_2	59.6 (5)	N1_4—C25'_4—C26'_4—C27'_4	−80.5 (13)
C26_2—C29_2—C30_2—C31_2	−60.8 (5)	O10'_4—C25'_4—C26'_4—C28'_4	−143.7 (16)
C26_2—C29_2—C30_2—C35_2	59.4 (5)	N1_4—C25'_4—C26'_4—C28'_4	42.2 (14)
C35_2—C30_2—C31_2—C32_2	−58.8 (5)	C29'_4—C26'_4—C27'_4—C32'_4	−59.6 (8)
C29_2—C30_2—C31_2—C32_2	61.3 (5)	C25'_4—C26'_4—C27'_4—C32'_4	−177.7 (7)
C30_2—C31_2—C32_2—C33_2	58.0 (6)	C28'_4—C26'_4—C27'_4—C32'_4	57.7 (8)
C30_2—C31_2—C32_2—C27_2	−60.8 (6)	C29'_4—C26'_4—C28'_4—C34'_4	59.7 (8)
C26_2—C27_2—C32_2—C31_2	60.1 (6)	C27'_4—C26'_4—C28'_4—C34'_4	−58.9 (8)
C26_2—C27_2—C32_2—C33_2	−59.5 (6)	C25'_4—C26'_4—C28'_4—C34'_4	177.8 (8)
C31_2—C32_2—C33_2—C34_2	−58.6 (6)	C27'_4—C26'_4—C29'_4—C30'_4	57.7 (8)
C27_2—C32_2—C33_2—C34_2	60.3 (5)	C25'_4—C26'_4—C29'_4—C30'_4	177.6 (8)
C32_2—C33_2—C34_2—C35_2	59.7 (5)	C28'_4—C26'_4—C29'_4—C30'_4	−60.9 (8)
C32_2—C33_2—C34_2—C28_2	−60.7 (5)	C26'_4—C29'_4—C30'_4—C35'_4	60.7 (8)
C26_2—C28_2—C34_2—C33_2	59.2 (5)	C26'_4—C29'_4—C30'_4—C31'_4	−58.5 (9)
C26_2—C28_2—C34_2—C35_2	−61.3 (5)	C35'_4—C30'_4—C31'_4—C32'_4	−59.4 (9)
C31_2—C30_2—C35_2—C34_2	60.0 (5)	C29'_4—C30'_4—C31'_4—C32'_4	60.2 (8)
C29_2—C30_2—C35_2—C34_2	−59.6 (5)	C26'_4—C27'_4—C32'_4—C33'_4	−57.6 (9)
C33_2—C34_2—C35_2—C30_2	−60.5 (5)	C26'_4—C27'_4—C32'_4—C31'_4	61.0 (9)
C28_2—C34_2—C35_2—C30_2	60.4 (5)	C30'_4—C31'_4—C32'_4—C27'_4	−61.1 (8)
O1_3—C1_3—C2_3—C3_3	−178.8 (4)	C30'_4—C31'_4—C32'_4—C33'_4	57.5 (9)
C1_3—C2_3—C3_3—C4_3	168.3 (4)	C27'_4—C32'_4—C33'_4—C34'_4	59.6 (9)
C2_3—C3_3—C4_3—C6_3	−177.6 (4)	C31'_4—C32'_4—C33'_4—C34'_4	−58.0 (9)
C2_3—C3_3—C4_3—C5_3	58.7 (5)	C32'_4—C33'_4—C34'_4—C35'_4	59.0 (9)
C3_3—C4_3—C6_3—C10_3	174.0 (3)	C32'_4—C33'_4—C34'_4—C28'_4	−60.4 (9)
C5_3—C4_3—C6_3—C10_3	−63.4 (5)	C26'_4—C28'_4—C34'_4—C33'_4	59.9 (8)
C3_3—C4_3—C6_3—C7_3	52.5 (5)	C26'_4—C28'_4—C34'_4—C35'_4	−58.7 (9)
C5_3—C4_3—C6_3—C7_3	175.2 (4)	C29'_4—C30'_4—C35'_4—C34'_4	−58.0 (9)
C4_3—C6_3—C7_3—C8_3	153.0 (4)	C31'_4—C30'_4—C35'_4—C34'_4	61.4 (9)
C10_3—C6_3—C7_3—C8_3	23.1 (4)	C33'_4—C34'_4—C35'_4—C30'_4	−60.7 (9)
C6_3—C7_3—C8_3—C9_3	4.3 (4)	C28'_4—C34'_4—C35'_4—C30'_4	57.4 (9)

### Hydrogen-bond geometry (Å, º)

**Table:**

*D*—H···*A*	*D*—H	H···*A*	*D*···*A*	*D*—H···*A*
O1_1—H1_1···O1_4	0.88 (2)	1.85 (3)	2.717 (5)	172 (6)
O2_1—H2_1···O3_3^i^	0.87 (2)	2.02 (3)	2.882 (4)	170 (5)
O1_2—H1_2···O1_3^ii^	0.88 (2)	1.86 (4)	2.686 (5)	154 (7)
O2_2—H2_2···O1_1	0.87 (2)	1.93 (3)	2.795 (4)	171 (5)
C22_2—H22*A*_2···O3_2	0.99	2.64	3.159 (7)	113
O1_3—H1_3···O3_2	0.88 (2)	1.76 (3)	2.627 (5)	167 (6)
O2_3—H2_3···O2_2	0.87 (2)	2.05 (3)	2.913 (4)	173 (5)
O1_4—H1_4···O3_1^ii^	0.88 (2)	1.92 (3)	2.796 (6)	174 (6)
O1_4—H1_4···O3′_1^ii^	0.88 (2)	2.20 (5)	2.881 (14)	135 (5)
O2_4—H2_4···O2_1^ii^	0.74 (5)	2.16 (5)	2.869 (4)	163 (6)
C22_4—H22*A*_4···O10_4	0.99	2.58	3.152 (13)	116
O1*W*—H1*W*···O2_4	0.86 (2)	2.16 (2)	3.001 (5)	170 (7)
O1*W*—H2*W*···O1_2	0.86 (2)	1.93 (3)	2.762 (6)	165 (8)

Symmetry codes: (i) *x*+1, *y*, *z*+1; (ii) *x*, *y*, *z*−1.
