# Supplementary figures and images for: (1S,3R)-N-{(3S,10S,12S,13R,17R)-12-Hy­droxy-17-[(R)-5-hy­droxy­pentan-2-yl]-10,13-di­methyl­hexa­deca­hydro-1H-cyclo­penta­[a]phenanthren-3-yl}adamantane-1-carboxamide 0.25-hydrate
Source: IUCrdata. 2022 Oct 11;7(Pt 10):x220947. doi: 10.1107/S2414314622009476 (PMC9638062; doi:10.1107/S2414314622009476)

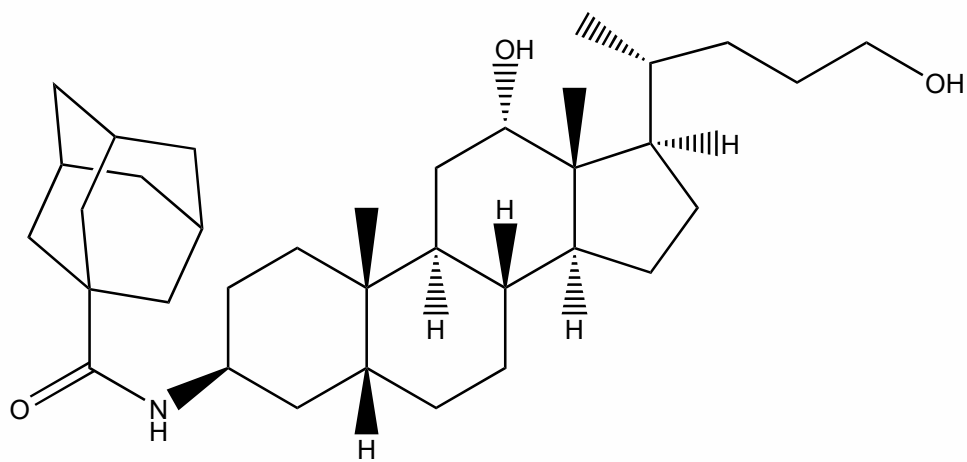

Supplement: Supplementary file 5 [file x-07-x220947-sup5.pdf]
